# Organic amendments alleviate early defoliation and increase fruit yield by altering assembly patterns and of microbial communities and enzymatic activities in sandy pear (*Pyrus pyrifolia*)

**DOI:** 10.1186/s13568-021-01322-5

**Published:** 2021-12-08

**Authors:** Yalong Kang, Xiangrui An, Yanwei Ma, Shaomin Zeng, Shangtao Jiang, Wenli Wu, Changyan Xie, Zhonghua Wang, Caixia Dong, Yangchun Xu, Qirong Shen

**Affiliations:** 1 Jiangsu Provincial Key Lab for Organic Solid Waste Utilization, National Engineering Research Center for Organic-Based Fertilizers, Jiangsu Collaborative Innovation Center for Solid Organic Waster Resource Utilization, Nanjing Agricultural University, Nanjing, 210095 China; 2 Fruit Research Institute, Research Centre for Engineering Technology of Fujian Deciduous Fruits, Fujian Academy of Agricultural Sciences, Fuzhou, 350013 China; 3 The Research Center of Agricultural Resources Environment and Soil Fertilizer, Huaian Academy of Agricultural Sciencess, Huaian, 223001 China; 4 Institute of Pomology, Jiangsu Academy of Agricultural Sciences, Nanjing, 210014 China

**Keywords:** Acid red soil, Pear tree, Early defoliation rate, Bioorganic fertilizer, Humic acid, Soil microbial activities, Co-occurrence network, Yield

## Abstract

Severe early defoliation has become an important factor restricting the development of the pear industry in southern China. However, the assembly patterns of microbial communities and their functional activities in response to the application of bioorganic fertilizer (BIO) or humic acid (HA) in southern China’s pear orchards remain poorly understood, particularly the impact on the early defoliation of the trees. We conducted a 3-year field experiment (2017–2019) in an 18-year-old ‘Cuiguan’ pear orchard. Four fertilization schemes were tested: local custom fertilization as control (CK), CK plus HA (CK-HA), BIO, and BIO plus HA (BIO-HA). Results showed that BIO and BIO-HA application decreased the early defoliation rate by 50–60%, and increased pear yield by 40% compared with the CK and CK-HA treatments. The BIO and BIO-HA application significantly improved soil pH, available nutrient content, total enzyme activity and ecosystem multifunctionality, and also changed the structure of soil bacterial and fungal communities. The genus *Acidothermus* was positively correlated with the early defoliation rate, while the genus *Rhodanobacter* was negatively correlated. Additionally, random forest models revealed that the early defoliation rate could be best explained by soil pH, ammonium content, available phosphorus, and total enzyme activity. In conclusion, application of BIO or BIO mixed with HA could have assembled distinct microbial communities and increased total enzyme activity, leading to significant improvement of soil physicochemical traits. The increased availability of soil nutrient thus changed leaf nutrient concentrations and alleviated the early defoliation rate of pear trees in acid red soil in southern China.

**Supplementary Information:**

The online version contains supplementary material available at 10.1186/s13568-021-01322-5.

## Introduction

Pears are the world’s third-largest consumer fruit. In recent years, early defoliation has become an important factor affecting the sustainable development of the pear industry in the red soil regions of southern China. Early defoliation usually happens from June to August, when the fruit quickly enlarges and until harvest, while the normal defoliation used to occur after late October. In some areas, such as pear orchards in Fujian, China, the early defoliation rate has been as high as 80% (Huang et al. 2010). Early defoliation affects nutrient accumulation in the trees, resulting in weakened growth, reduced photosynthesis, and ultimately a decline in fruit quality and yield (Huang et al. 2010).

Farmers prefer using large amounts of chemical fertilizers to produce profitable yields. This has been at best a short-term solution, the intensive application of fertilizers has resulted in high leaf nitrogen (N) content, and low leaf medium and trace element, which is an important factor in the susceptibility to leaf diseases (Dordas 2008; Sete et al. 2019). For example, Fu et al. (2019) found that the anthracnose *Colletotrichum* spp.*,* which infects pear leaves, is the most significant factor in severe early defoliation of pear trees in southern China. That are associated with intensified application of chemical fertilizers (Dong et al. 2012; Jiang et al. 2020a; Ma et al. 2018), such as increased soil acidification, deplete soil fertility, and inhibit soil microbial activity (Dong et al. 2012; Gu et al. 2019; Jiang et al. 2020a; Ma et al. 2018). It seriously weakens the growth and vitality of pear trees, which might be also an essential factor for the early defoliation of pear trees in southern China. According to a survey by Huang et al. (2010), the degree of early defoliation rate of pear trees in orchards with high soil organic matter content and soil pH above 5.0 is lower, indicating that improving soil physicochemical traits may reduce the early defoliation rate.

Organic amendments include humic acid (HA) (Li et al. 2020b; Zaller 2006) and organic fertilizer (Jiang et al. 2020b; Liu et al. 2019; Ma et al. 2018) have been widely used to maintain or increase soil fertility, microbial activity, and improve fruit yield in orchards. The use of organic amendments combined with chemical fertilizers, or the partial replacement of chemical fertilizers with organic fertilizers, is viewed as a feasible way to increase crop yields in the short term, and increase soil organic matter in the long term (Ji et al. 2020; Li et al. 2017; Wei et al. 2016). Although the commercial pear orchards in southern China have adopted the strategy of combining organic with chemical fertilizers for four years (from 2013 to 2016), the early defoliation rate of pear trees has not significantly changed, and yield have indeed declined.

Bioorganic fertilizer (BIO) is an organic amendment prepared by secondary solid-state fermentation of organic materials, with the addition of specific functional microbial strains. It has been widely used in apple (Wang et al. 2016), banana (Shen et al. 2019), pepper (Yu et al. 2019), and kiwi (Liu et al. 2020c) orchards to improve soil biological fertility, and restructure soil microflora. Soil microbes have an enormous impact on soil fertility, ecosystem sustainability, as well as plant health and growth (Gu et al. 2019; Li et al. 2021; Ma et al. 2018; Zhang et al. 2021). However, there is a lack of information on how BIO and HA affect the early defoliation rate, the soil microbial activities and community composition, and their relationships of sandy pear orchards in the red soil regions of southern China.

Microbial co-occurrence networks are widely used to reveal the associations between network members and provide insights into the function of the microbiome (Faust and Raes 2012). Zheng et al. (2018) have found that organic amendments could increase the negative correlations within the co-occurrence network of rhizosphere microbial communities of apple trees, implying the increase in competition among rhizosphere microbial taxa. Liu et al. (2020c) reports that bioorganic fertilizer could increase the complexity and stability of the soil microbial co-occurrence network in kiwi orchards. These studies show that the application of organic amendments can lead to changes in specific soil microbial co-occurrence networks. It is still unknown though, how BIO and HA affect the soil microbial co-occurrence network of sandy pear trees in southern China.

Herein, we conducted a three-year field experiment in a commercial pear orchard in Fujian province, China. Orchards in this area have suffered severe early defoliation rate for more than ten years. The main purpose of this study was to determine if bioorganic fertilizer (BIO) and humic acid (HA) could alleviate the early defoliation rate of pear trees in in comparison with conventionally used fertilizers. We also sought to determine the impact of each type of fertilizer on leaf nutrient concentration, soil enzyme activities, microbial community structure and their co-occurrence network, and pear yield. Our hypothesis is that single application of BIO, HA or their combined application will improve the total enzyme activity, and shift the soil microbial community structure and their co-occurrence network, and thus change the leaf nutrient state and alleviate the early defoliation rate of pear trees in an acid red soil.

## Materials and methods

### Experimental site and design

The study was conducted from October 2016 to July 2019 in a commercial pear orchard in Jianning County, Fujian Province, China (34°04′ N, 108° 10′ E). The site is a mountain orchard and the climate is subtropical monsoon, and it has an average annual temperature of approximately 16.9 ℃ and an average annual precipitation of 1850 mm. The soil type is red loam and the texture is clay. Before the experiment, the soil properties were: pH 4.45, soil organic matter (SOM) 19.7 g kg^−1^, total nitrogen (TN) 0.49 g kg^−1^, available phosphorus (AP) 37.7 mg kg^−1^, available potassium (AK) 149.0 mg kg^−1^. The orchard is an 18-year-old ‘Cuiguan’ pear orchard belonging to the Lvyuan Fruit Industry Co., Ltd., its area is 1800 m^2^, with a density of 600 trees ha^−1^ (4 m distance between trees and 4 m between rows). The study was conducted using a randomized complete block design with four treatments and three replicates per treatment; each plot, or block, contained 9 pear trees. The treatments were as follows: (1) CK, conventional fertilization, i.e. commercial organic fertilizer (organic matter content ≥ 50%, total nutrients ≥ 5.0%) 6000 kg ha^−1^ mixed with chemical fertilizer (N:P_2_O_5_:K_2_O = 24:8:10) 6000 kg ha^−1^; (2) CK-HA, commercial organic fertilizer and chemical fertilizer as above, combined with HA (N:P_2_O_5_:K_2_O = 12:5:3, humic acid 30%, pH 6.0) 240 kg ha^−1^; (3) BIO, bioorganic fertilizer (N + P_2_O_5_ + K_2_O ≥ 8%, organic content ≥ 40%, functional microorganisms *Bacillus amyloliquefaciens* SQR9 about 1 × 10^8^ CFU g^−1^ dry weight) 12,000 kg ha^−1^; (4) BIO-HA, BIO as above, and HA 240 kg ha^−1^. In accordance with the local fertilization protocol, all of commercial organic fertilizer, BIO, and three-fifths of the chemical fertilizer applied as a base fertilizer in mid-November of each year (2016 to 2018). The remaining chemical fertilizer was applied as a top dressing during the budding stage (mid-to-late March) and fruit expanding stage (mid-June). For CK-HA and BIO-HA treatments, humic acid was applied in mid-May (young fruit stage) by irrigation; specifically, 0.4 kg of humic acid mother liquor was diluted 100 times with tap water, mixed thoroughly, then applied around each tree approximately 100 ~ 140 cm from the trunk. For CK and BIO treatments the irrigation was with water only. See Additional file 1: Table S1 for the detailed fertilization schedule. All other aspects of field management were carried out according to the local traditional farming methods. Due to the contingency and uncertainty of the test results in the first year (2017), we began to investigate the early defoliation rate and other related parameters from 2018 to 2019.

### Early defoliation rate

The early defoliation of treated pear trees was measured from April 20th to July 10th, in 2018 and 2019. Six pear trees were randomly selected in each plot; on these trees, three 2–3-year-old branches growing in different directions along the radial axis of the trunk were randomly selected for leaf counting. The selected trees and branches were labeled with red spray paint. Leaves were manually counted starting from the healing site of the graft to the apical growing point. N_0_ is the initial leaf count on April 20, N_1_ is the leaf count on May 20 and so on. The calculation for the early defoliation rate (ED) is:

1 $${\text{ED }}\left( \% \right) \, = { 1}00 \, *\left( {{1} - {\text{N}}_{{\text{i}}} /{\text{ N}}_{0} } \right)$$

where, N_i_ represents the number of leaves in the i-th survey (i = 1, 2, …, n).

### Yield

Pear yield was measured on July 10, in 2018 and 2019 at the fruit maturity stage. Twenty picked fruits from every tree in each plot, similar in size, with no evident mechanical or insect damage, or disease were weighed. The yield per tree was calculated using the total number of fruits per tree times the average weight of the twenty selected fruits from that tree. Total yield per hectare (Mg ha^−1^) was calculated based on the number of trees per hectare.

### Leaf collection and trait measurement

Just after the last leaf count, 100 mature leaves from unmarked branches neighboring the marked branches, were randomly selected and marked. The relative chlorophyll content (SPAD) of these leaves was measured using a portable SPAD-502 (Konica Minolta). The leaves were then picked, placed in zip-lock bags, and quickly transported to the laboratory. The area of each leaf was then measured (LA), then leaves were heated for 30 min at 105 ℃, dried for 72 h at 65 ℃, then weighed to determine dry mass (g). The dried leaves were fully triturated by passage through a 100-mesh sieve, and stored in zip-lock bags at 25 ℃ until further use. A Vario TOC (Elementar Company, Hanau, German) was used to measure the carbon (C) and nitrogen (N) concentrations of the leaves. The phosphorus (P), potassium (K), calcium (Ca), magnesium (Mg), iron (Fe), manganese (Mn), zinc (Zi), and copper (Cu) concentrations were quantified using an ICP-AES (Iris Advantage 1000, Thermo Jarrell Ash, Franklin, MA, USA) (Du et al. 2017).

### Soil sampling and physicochemical analysis

According to the method modified from Bonilla et al. (2015) soil samples were taken on July 11 to 13, just after fruit harvest, in 2018 and 2019. Soil was sampled from around each of the six marked pear trees in each plot. At 100–140 cm horizontally away from the trunk, soil was sampled from a depth of approximately 6–40 cm at four equidistant positions around the trunk (the upper ~ 5 cm of soil had been removed before sampling). The four samples from each tree were combined and represent one replicate. In all, six replicates were collected from each treatment group, for a total of 24 soil samples. All samples were collected into sterile zip-lock bags and stored at 4 °C until further use. Soil samples were sieved (2 mm) to remove aboveground plant materials, roots, and stones then divided into three subsamples: one was air-dried for soil physicochemical analysis, one was stored at 4 °C for soil microbial activity analysis, and one was stored at − 80 °C for DNA extraction and MiSeq sequencing.

### Soil enzyme activities

The MUB (4-methylumbelliferyl)-linked model substrates method was used to determine the soil enzyme activities related to carbon, nitrogen, and phosphorus cycles (DeForest 2009). Enzyme activity is expressed as nmol h^−1^ g^−1^ soil. Geometric mean enzyme activity (GMEA) and total enzyme activity (Et) were used to reflect the effect of soil amendments on the overall soil enzyme activity and fertility levels (García-Ruiz et al. 2008). These were calculated as follows:

2 $${\text{GMEA }} = \left( {{\text{X}}_{{1}} \cdot {\text{ X}}_{{2}} \cdot {\text{ X}}_{{3}} \cdot {\text{X}}_{{\text{n}}} } \right)^{{1/{\text{n}}}}$$

where X represents the activity of an enzyme, and n represents the number of types of that enzyme.

3 $${\text{Et = ~}}\sum\limits_{{{\text{i = 1}}}}^{{\text{n}}} {{\text{Xi/}}} \overline{{\text{x}}}$$

where X_i_ is the measured value of the activity of the i-th enzyme and $\overline{{\text{x}}}$ is the average value of the activity of the same type of enzyme. The functions of specific enzymes are shown in Additional file 1: Table S2.

In an effort to quantify soil biodiversity, ecosystem multifunctionality (EMF) indices are often used in the study of soil ecosystems. These indices are a helpful tool for understanding complex and interactive processes (Luo et al. 2018). To obtain a value for EMF, we first standardized the value of each soil enzyme activities (seen details in Additional file 1: Table S2) to obtain their Z-scores (Delgado-Baquerizo et al. 2016), and then average of the standardized values of the nine enzyme activities under the same treatment was calculated.

### DNA extraction and high-throughput sequencing

Genomic DNA was extracted from 0.5 g of soil using a MoBioPowerSoil™ DNA Isolation Kit (MoBio Laboratories, Carlsbad, USA). The quantity and quality of the extracted DNA were measured using a Nanodrop ND-2000 UV–VIS spectrophotometer (NanoDrop Technologies, Wilmington, USA). The V4 region of the bacterial 16S rRNA was amplified using the primers 515F (5′-GTGYCAGCMGCCGCGGTAA-3′) and 806R (5′-GGACTACNVGGGTWTCTAAT-3′) (Walters et al. 2016). The fungal ITS2 region was amplified using the primers ITS2-F (5′-GCATCGATGAAGAACGCAGC-3′) and ITS2-R (5′-TCCTCCGCTTATTGATATGC-3′) (Zuo et al. 2018). PCR amplification conditions and high-throughput sequencing are as described in Gu et al. (2019) and Ji et al. (2020), except that we used the Illumina HiSeq 2500 platform (Guangdong MAGIGENE Biotechnology Co. Ltd, China) to sequence the bacterial and fungal amplicon library.

### Sequence data analysis

The analysis of sequenced data was as described in Fan et al. (2020). Briefly, sequences were processed with the QIIME software package (Quantitative Insights Into Microbial Ecology) and UPARSE pipeline. The reads were filtered by QIIME quality filter. The sequences retained for each sample, referred to as clean paired sequences, were analyzed following the UPARSE pipeline to pick up operational taxonomic units (OTUs) through making OTU table. Sequences with a quality score lower than 0.5 or length shorter than 200 nt and singletons were discarded, and the retained sequences were assigned to OTUs at 97% similarity, and chimeras were filtered. Then the most abundant sequence from each OUT was selected as a representative sequence for that OTU and taxonomically annotated with the SILVA (bacteria) and UNITE (fungi) databases. The raw sequence data reported in this paper have been deposited in the Genome Sequence Archive of the BIG Data Center, Chinese Academy of Sciences, under accession code PRJCA005679 and PRJCA005685.

### Co-occurrence network analysis of bacteria and fungi

Using a Spearman correlation matrix, a co-occurrence network was derived to assess the relationships between soil microbial taxa. Before constructing the network, data from BIO and BIO-HA were combined and are referred to as + BIO, while CK and CK-HA data were combined and are identified as -BIO. This preprocessing step allowed us to clarify the influence of BIO application on bacterial and fungal network interactions. Similarly, to clarify the influence of HA on network interactions, we combined CK-HA and BIO-HA, identified herein as + HA; CK and BIO were combined and are identified as -HA. OTUs with a relative abundance of less than 0.01% and an occurrence frequency of less than 6 out of 8 data columns were deleted to reduce rare OTUs in the data set. Spearman correlation and network properties were calculated using the psych and igraph packages in R (version 3.5.1). After adjusting for Benjamini-Hochberg's false discovery rate, we retained results with an absolute r value greater than 0.6 and a p value less than 0.05. The network was visualized using “gephi” 0.9.2 (https://gephi.org/) and network topological parameters were calculated (Benjamini and Hochberg 1995).

### Statistical analysis

Data were preprocessed using Excel 2010. All results were reported as means ± standard errors (SE) for the six replicates. SPSS 25.0 (IMB Corp., Armonk, USA) was used to conduct one-way ANOVA of the soil physical and chemical properties, microbial diversity and activity, leaf nutrient content, early defoliation rate, and yield. The Bray–Curtis distance-based non-metric multidimensional scale (NMDS) was used in R (v3.5.0) to visualize microbial β-diversity in the samples. Permutation multivariate analysis of variance (PERMANOVA) and similarity analysis (ANOSIM) based on the Bray–Curtis distance matrix were used in the vegan package in R (v3.5.0) to evaluate the significance of differences between soil microbial communities under different fertilization treatments. Relationships among parameters were analyzed using the Spearman correlation in R. The threshold for statistical significance was defined as a P value < 0.05. The classification random forest analysis was employed to identify the most important and credible predictors of early defoliation rate among various variables in the randomForest package in R (Breiman 2001).

## Results

### Soil physicochemical properties

Organic amendments (BIO and BIO-HA) application significantly (*P* < *0.05*) increased soil pH about 0.7 units compared with those in the CK and CK-HA treatments in both 2018 and 2019. Significant differences (*P* < *0.05*) in soil organic matter (SOM) were observed between organic amendments treatments and CK treatment in 2018 and 2019. Compared with CK treatment, the addition of organic amendments increased the content of available nutrients in pear orchard soil from 2018 to 2019. In 2019, the NH_4_^+^-N and NO_3_^−^-N content of BIO and BIO-HA treated soil was 110% to 210% greater than CK treated soil (*P* < *0.05*) (Table 1).

**Table 1:** Effect of organic amendments on the physicochemical characteristics of the pear orchard acid red soil

	Treatments			
	CK	CK-HA	BIO	BIO-HA
Soil properties in 2018 yr				
pH	4.6 (0.21) b	4.8 (0.13) b	5.3 (0.08) a	5.4 (0.13) a
SOM g kg^−1^	19.4 (0.72) b	20.1 (0.37) ab	22.7 (1.47) a	22.1 (1.92) a
TN g kg^−1^	0.9 (0.06) b	1.2 (0.11) ab	1.3 (0.20) ab	1.5 (0.11) a
NH_4_^+^-N mg kg^−1^	17.5 (3.00) b	28.1 (2.53) a	27.7 (1.51) a	35.9 (4.59) a
NO_3_^−^-N mg kg^−1^	35.6 (2.92) b	36.6 (2.12) b	28.8 (1.38) b	48.6 (5.29) a
AP mg kg^−1^	91.4 (8.27) b	115.2 (11.40) b	158.7 (7.7) a	181.2 (19.03) a
AK mg kg^−1^	123.7 (17.87) b	148.3 (21.1) b	222.9 (22.82) a	281.7 (22.65) a
C/N	12.2 (0.92) a	10.3 (1.15) a	10.4 (1.99) a	8.7 (0.83) a
Soil properties in 2019 yr				
pH	4.7 (0.16) a	4.9 (0.07) a	5.4 (0.10) b	5.5 (0.12) b
SOM g kg^−1^	20.9 (1.56) b	21.3 (0.91)a	23.7 (1.59) a	24.5 (2.37) a
TN g kg^−1^	1.0 (0.08) b	1.3 (0.24) ab	1.3 (0.19) ab	1.6 (0.10) a
NH_4_^+^-N mg kg^−1^	21.0 (2.30) b	32.0 (2.27) b	44.2 (3.97) a	56.6 (4.79) a
NO_3_^—^N mg kg^−1^	27.9 (1.69) d	46.9 (4.27) c	68.1 (4.31) b	87.0 (5.72) a
AP mg kg^−1^	84.9 (6.08) b	104.9 (5.58) b	166.2 (26.85) a	187.2 (22.97) a
AK mg kg^−1^	152.9 (25.47) b	198.4 (18.61) b	265.9 (32.42) ab	348.0 (55.90) a
C/N	12.3 (1.20) a	10.6 (1.94) a	10.7 (1.05) a	8.9 (0.32) a

*CK* conventional fertilization, *HA* humic acid amendment, *CK-HA* conventional fertilization combined with HA, *BIO* bio-organic fertilizer, *BIO-HA* mixed application of BIO and HA, *SOC* soil organic carbon, *TN* total nitrogen, *NH*_*4*_^*+*^*-N* ammonium nitrogen, *NO*_*3*_^*−*^*-N* nitrate-nitrogen, *AP* available phosphorus, *AK* available potassium, *C/N* ration of soil carbon content to nitrogen content

Each value represents the mean (n = 6), and standard error values are indicated with ±

Different letters indicate significant difference (*P* < *0.05*) in every column among the four treatments as determined by Fisher’s least significant difference test (LSD) at α = 0.05

### Leaf nutrient

Compared with CK and CK-HA treatments, BIO and BIO-HA decreased the leaf N concentration about 5–30% from 2018 to 2019. Particularly in 2019, the effect was the most significant (*P* < *0.05*). Soils treated with BIO and BIO-HA had significantly (*P* < *0.05*) increased K concentration in 2018, but there were no significant change in 2019 compared with the CK. Leaf Ca concentration were significantly (*P* < *0.05*) accumulated by 52–77% under the BIO and BIO-HA treatments in comparison to the CK from 2018 to 2019. For leaf Fe concentration, BIO and BIO-HA significantly (*P* < *0.05*) enhanced it by 85% and 135% in 2019, respectively, compared with those in the control. At the end of the study (in 2019), compared with CK treatment, BIO-HA treatment significantly (*P* < *0.05*) enriched leaf Mg, Mn and Cu concentration about 32–117%. However, there were no significant differences in leaf C, P and Zn contents between the treatments (Additional file 1: Table S3).

### Early defoliation rate and yield of pear trees

**Fig. 1:**
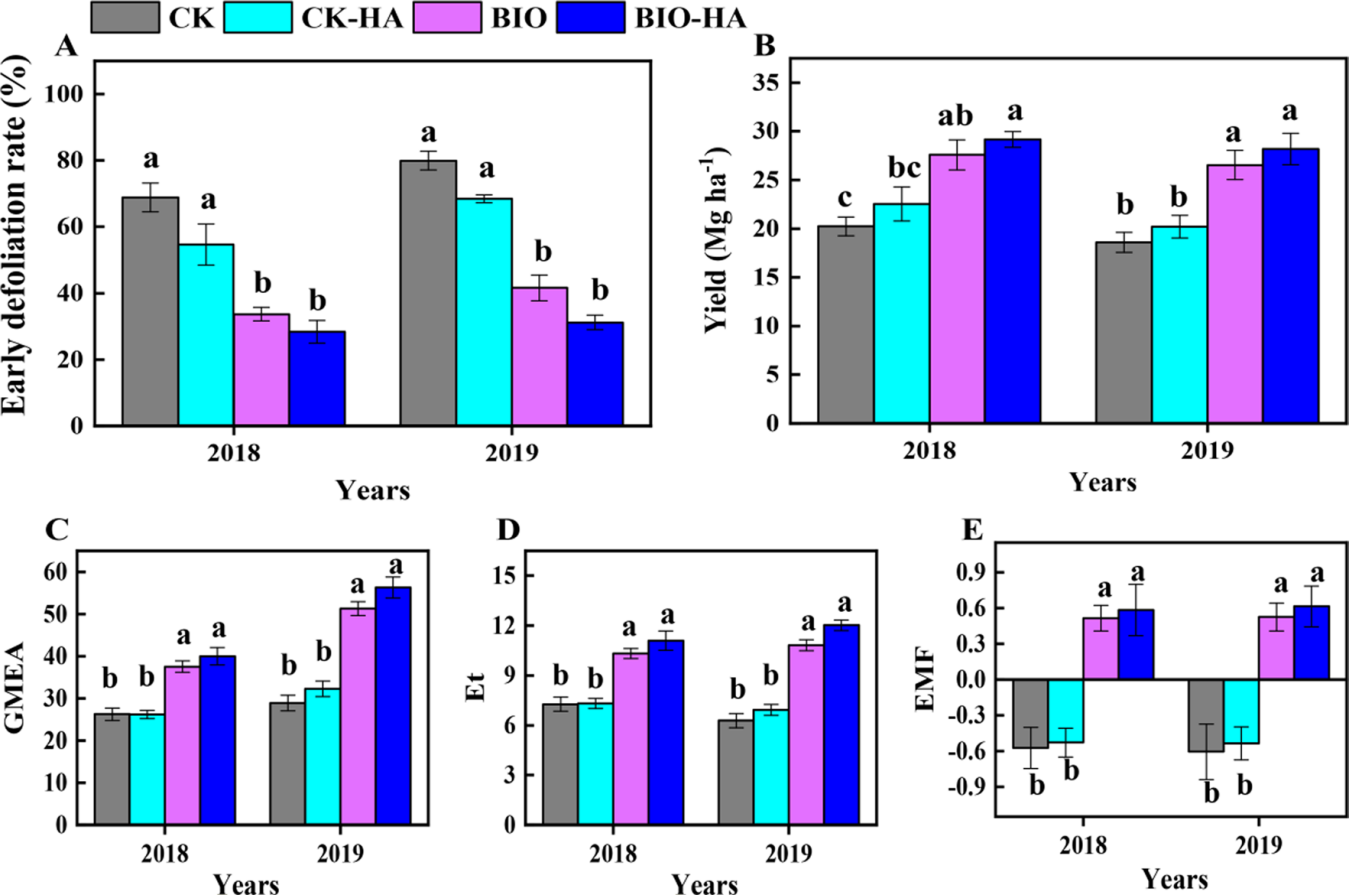
Effect of organic amendments on the early defoliation rate (**A**) and yield (**B**), and geometric mean enzyme activity (**C**), total soil enzyme activity (**D**) and ecosystem multifunctionality (**E**) of pear trees in 2018 and 2019. GMEA, geometric mean enzyme activity; Et, total enzyme activity; EMF, ecosystem multifunctionality. CK, conventional fertilization; CK-HA, conventional fertilization combined with humic acid; BIO, bioorganic fertilizer; BIO-HA, combined application of BIO and HA. Different lowercase letters indicate significant difference at 0.05 levels (LSD, P < 0.05). The error bars are the standard errors (n = 6)

In 2018 to 2019, BIO and BIO-HA treated trees had a significantly (*P* < *0.05*) reduced early defoliation rate; at the fruit maturity stage the early defoliation rate was 50–60% less than CK treated trees (Fig. 1A). The yield from BIO and BIO-HA treated trees was about average 40% greater than the yield from the CK and CK-HA treated trees in 2018–2019 (Fig. 1B). This is positively correlated to the decrease in early defoliation rate, and the increase in the SPAD and leaf area (Additional file 1: Fig. S1 and S2) in the BIO and BIO-HA treated trees.

### Soil enzyme activities

In 2018, soils treated with BIO or BIO-HA had no significant effects on αG and ACP activities, but in 2019, the effect was the most significant (*P* < *0.05*). At the end of the study (2019), BIO and BIO-HA had significantly increased enzyme activities related to carbon, nitrogen, and phosphorus (βG, βX, CBH, LAP, ACP and NAG) in comparison to the CK. There were no significant differences in the activities of PeO and PhO in both 2018 and 2019 (Additional file 1: Fig. S3). In addition, GMEA, Et, and EMF were significantly higher in BIO and BIO-HA treated soil than those in CK and CK-HA treatments (Fig. 1C–E). Spearman correlation analysis revealed a significantly negative correlation between early defoliation rate and GMEA, Et and EMF (Additional file 1: Fig. S1). These data demonstrate that the organic amendments not only improved the fertility and ecosystem multifunctionality of the pear orchard soil, but are important factors in reducing the early defoliation rate of pear trees.

### Soil microbial diversity and community composition

**Fig. 2:**
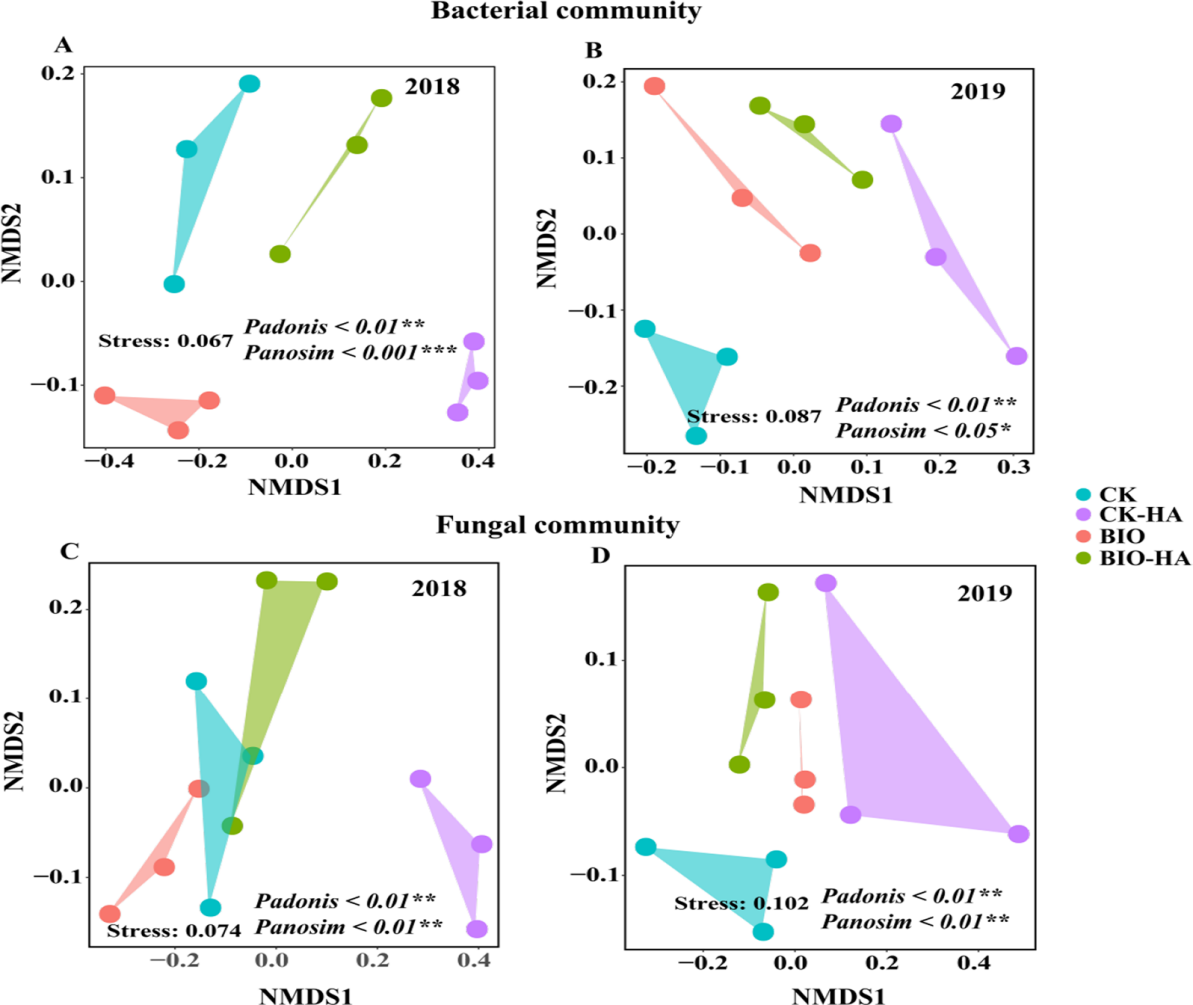
Bacterial (**A** and **B**) and fungal (**C** and **D**) communities in soil treated with organic amendments. CK, conventional fertilization; CK-HA, conventional fertilization combined with humic acid; BIO, bio-organic fertilizer; BIO-HA, combined application of BIO and HA. *P < 0.05; **P < 0.01; ***P < 0.001

The alpha diversity (chao1 and Shannon) of soil bacteria and fungi was only the highest in CK-HA treatments in 2018, but no significant differences were found among all treatments in 2019 (Additional file 1: Fig. S4). In 2018, NMDS analysis based on UniFrac distance showed that the fungal community structure of CK-HA treated soil was significantly different from the other treated soils. In 2019, after 3 years of treatment, organic amendments had significantly changed the bacterial and fungal community structure of the tested soils (*Padonis* and *Panosim* < 0.05) (Fig. 2).

**Fig. 3:**
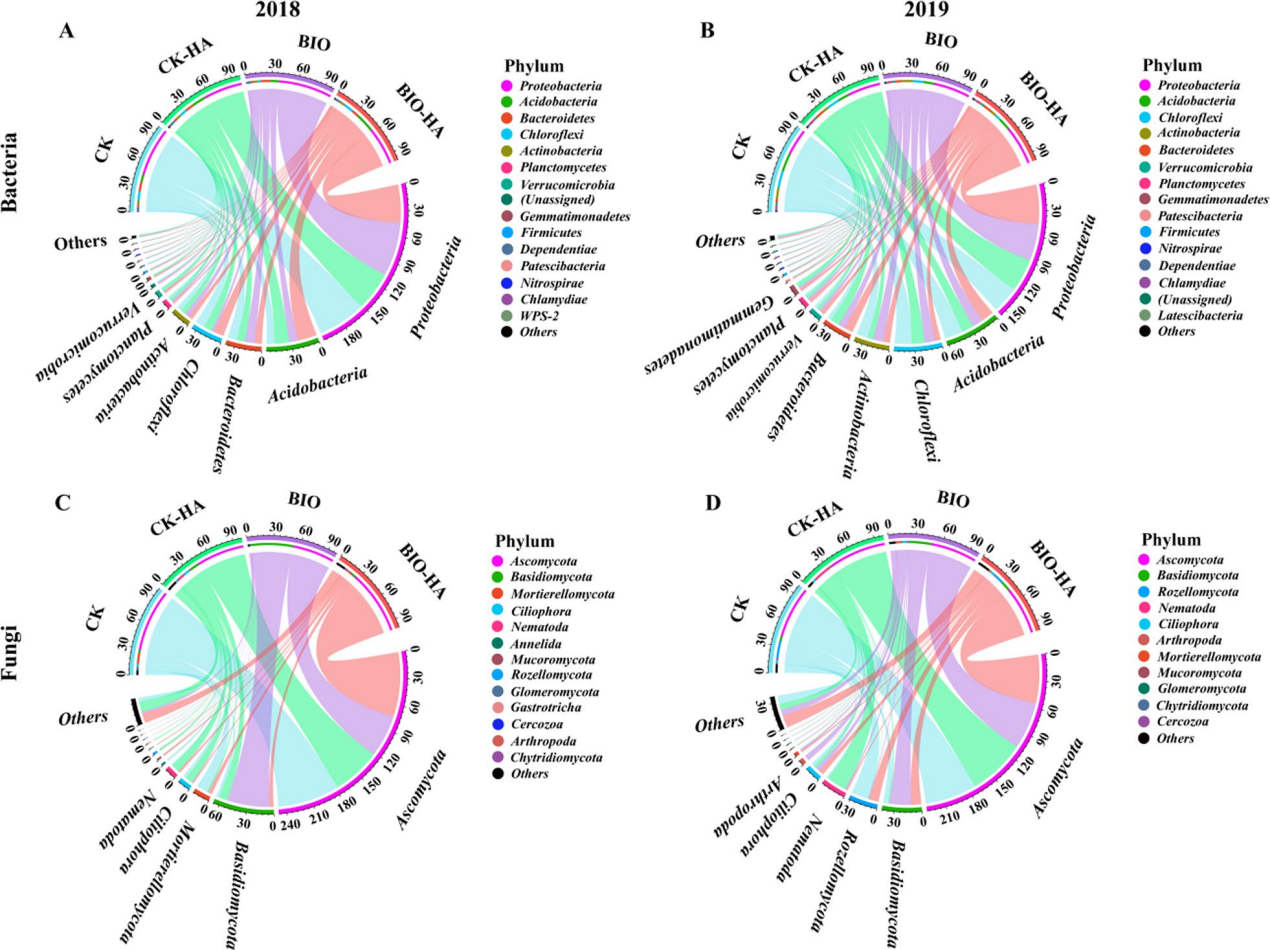
Relative abundance of the 15-dominant bacterial (**A** and **B**) and fungal (**C** and **D**) phyla 2018–2019. The change in the width of the color band indicates the change in relative abundance. CK, conventional fertilization; CK-HA, conventional fertilization combined with humic acid; BIO, bio-organic fertilizer; BIO-HA, combined application of BIO and HA

In the sandy pear orchard soil treated with organic amendments, *Proteobacteria* (30–60%) and *Acidobacteria* (9–22%) were the most dominant bacterial phyla, while *Ascomycota* (48–71%) and *Basidiomycota* (2–48%) were the most dominant fungal phyla (Fig. 3 and Additional file 1: Fig. S5). For bacteria, compared with CK, CK-HA treated soils had a significantly increased the relative abundance of *Acidobacteria* in 2018, but decreased in 2019. In BIO treated soils, the relative abundance of *Chlamydiae* was significantly increased compared with CK treated soil from 2018 to 2019. Relative abundance of *Chloroflexi* and *Actinobacteria* were significantly decreased by BIO-HA treated soils compared to CK in 2019. For fungi, CK-HA treatment significantly inhibited the relative abundance of *Mucoromycota* and *Glomeromycota* compared with CK in 2019. Additionally, relative abundance of *Glomeromycota* was significantly lower in BIO treated soil than those in CK treated soil (Fig. 3 and Additional file 1: Fig. S5).

**Fig. 4:**
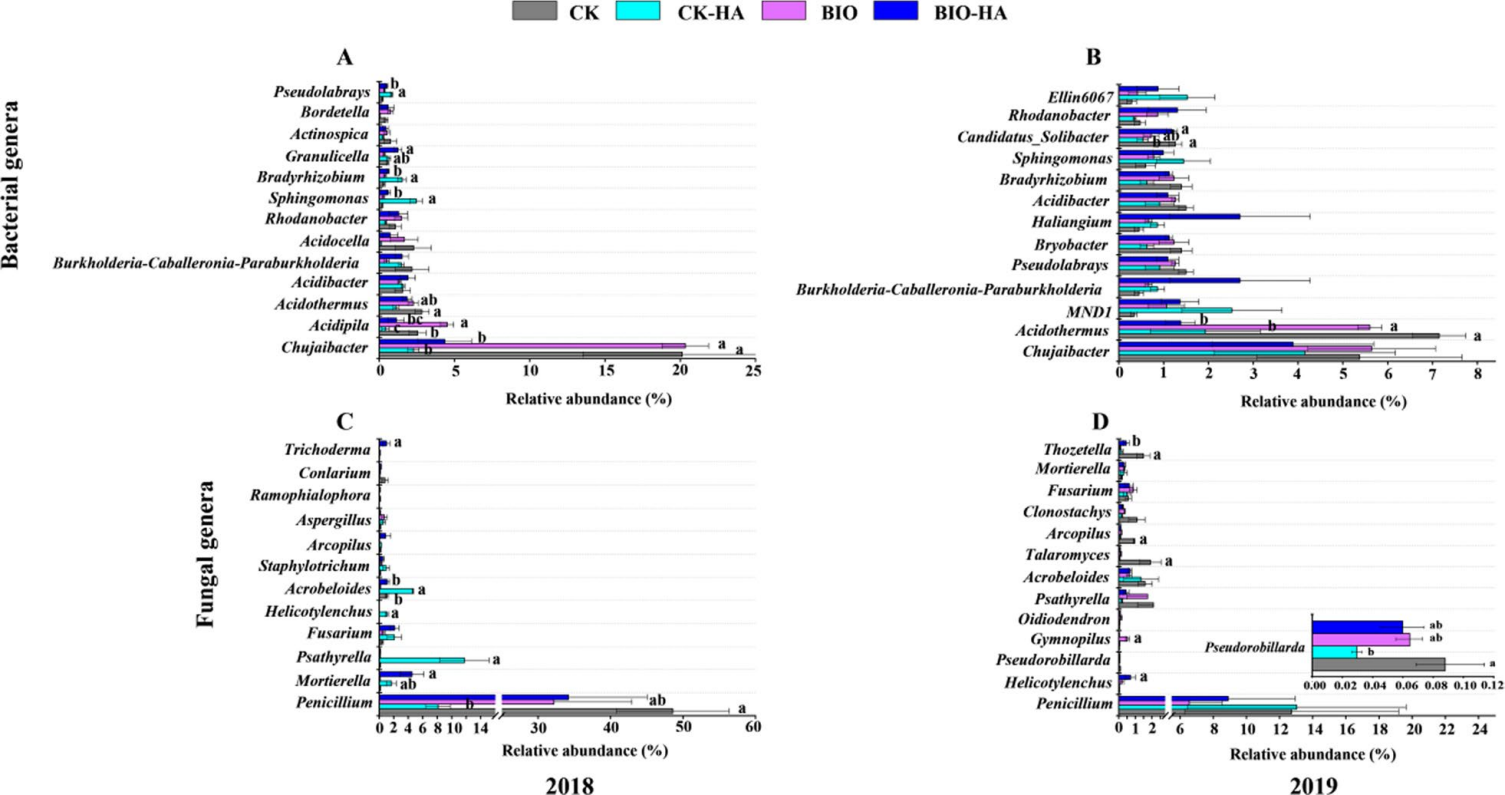
Relative abundance of the 15-dominant bacterial (**A** and **B**) and fungal (**C** and **D**) genera 2018–2019. CK, conventional fertilization; CK-HA, conventional fertilization combined with humic acid; BIO, bio-organic fertilizer; BIO-HA, combined application of BIO and HA. Different lowercase letters indicate significant difference at 0.05 levels (LSD, P < 0.05). The error bars are the standard errors (n = 3)

At the bacterial genus level, compared with CK treated soils, CK-HA treatment increased the relative abundance of *Sphingomonas* in both 2018 and 2019, but decreased the relative abundance of *Bradyrhizobium* in 2019; BIO-HA treatments increased the relative abundance of *Burkholderia-Caballeronia-Paraburkholderia*, *Haliangium*, *Sphingomonas*, and *Rhodanobacter* in 2019. At the fungal genus level, compared with CK treated soils, relative abundance of *Psathyrella* enriched by CK-HA treatment in 2018, but then reduced in 2019. BIO and BIO-HA treatment significantly increased the relative abundance of *Gymnopilus* and *Helicotylenchus* compared with CK treatment in 2019, respectively (Fig. 4).

### Effect of organic amendments on bacterial and fungal co-occurrence patterns

**Fig. 5:**
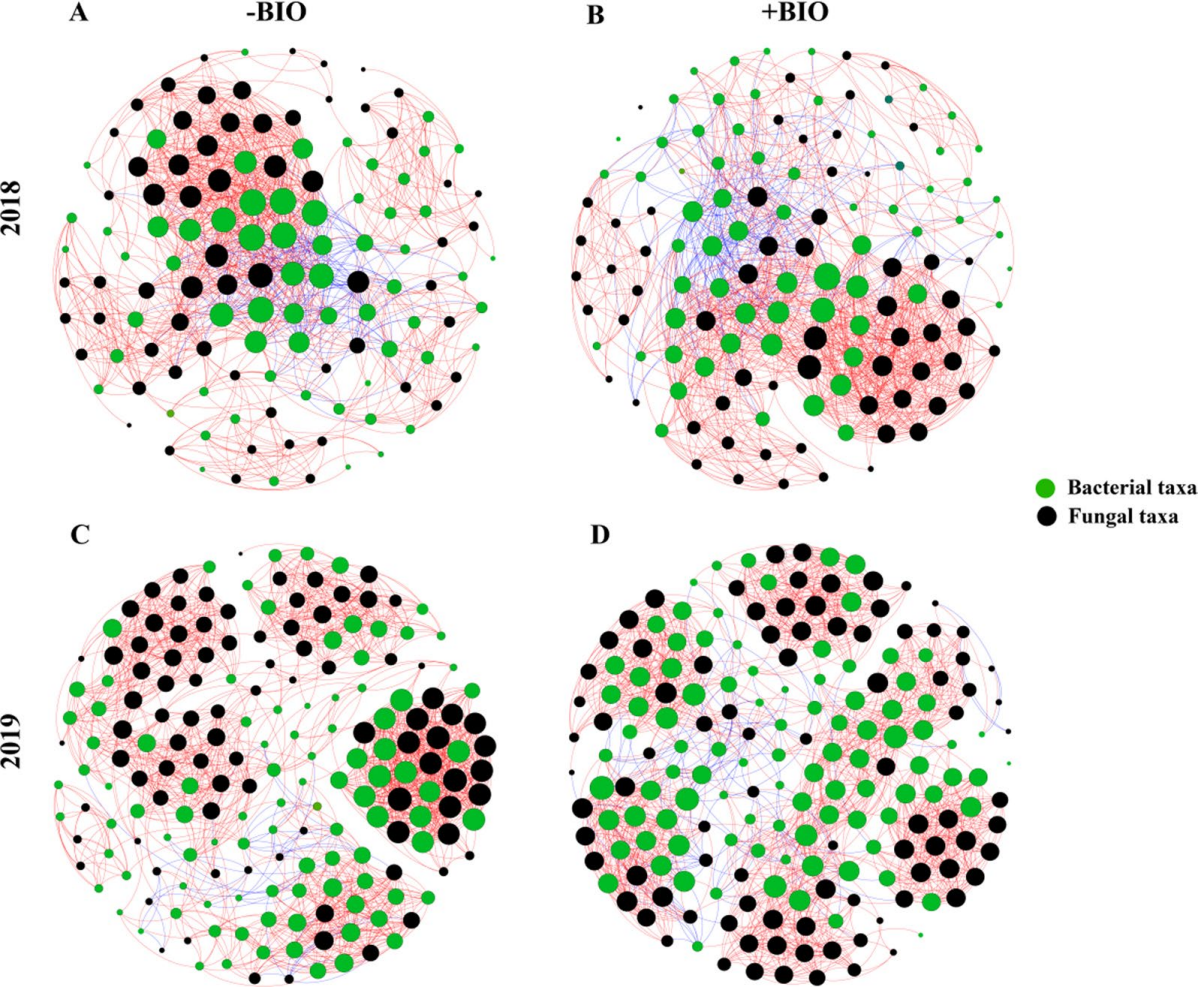
Network analysis revealing the co-occurrence pattern between bacterial and fungal OTUs in -BIO (CK and CK-HA, **A** and **C**) and + BIO (BIO and BIO-HA, **B** and **D**) treated soils, 2018 and 2019. Green and black colored nodes signify corresponding OTUs assigned to bacterial and fungal major phyla, respectively. Red lines and blue lines represent strong positive linear (r > 0.6) and strong negative linear (r < − 0.6) and relationships, respectively. The size of each node is proportional to the number of connections (degree)

Application of BIO and HA has different effects on the co-occurrence network of bacterial and fungal communities in the treated soils. The number of bacterial nodes accounts for about 51% and the number of fungal nodes accounts for about 49% in all the treated soil (Fig. 5, Additional file 1: Fig. S6 and Table S4). BIO (+ BIO vs -BIO) reduced the number of positive links between bacterial and fungal communities (from 434 to 401), while HA (+ HA vs -HA) reduced the number of negative links between bacterial and fungal communities (from 75 to 32). The addition of organic amendments (BIO and HA) reduced the average path length and network diameter of the microbial co-occurrence network. + BIO reduced the clustering coefficient of the co-occurrence network of bacteria and fungi, but increased network centralization. The addition of HA however, enhanced the compactness of the co-occurrence network of bacteria and fungi, and the concentration of nodes in the whole network towards the center was decreased (Fig. 5, Additional file 1: Fig. S6 and Table S4).

Relationships between soil chemistry, microbial taxa, and early defoliation rate and yield.

**Fig. 6:**
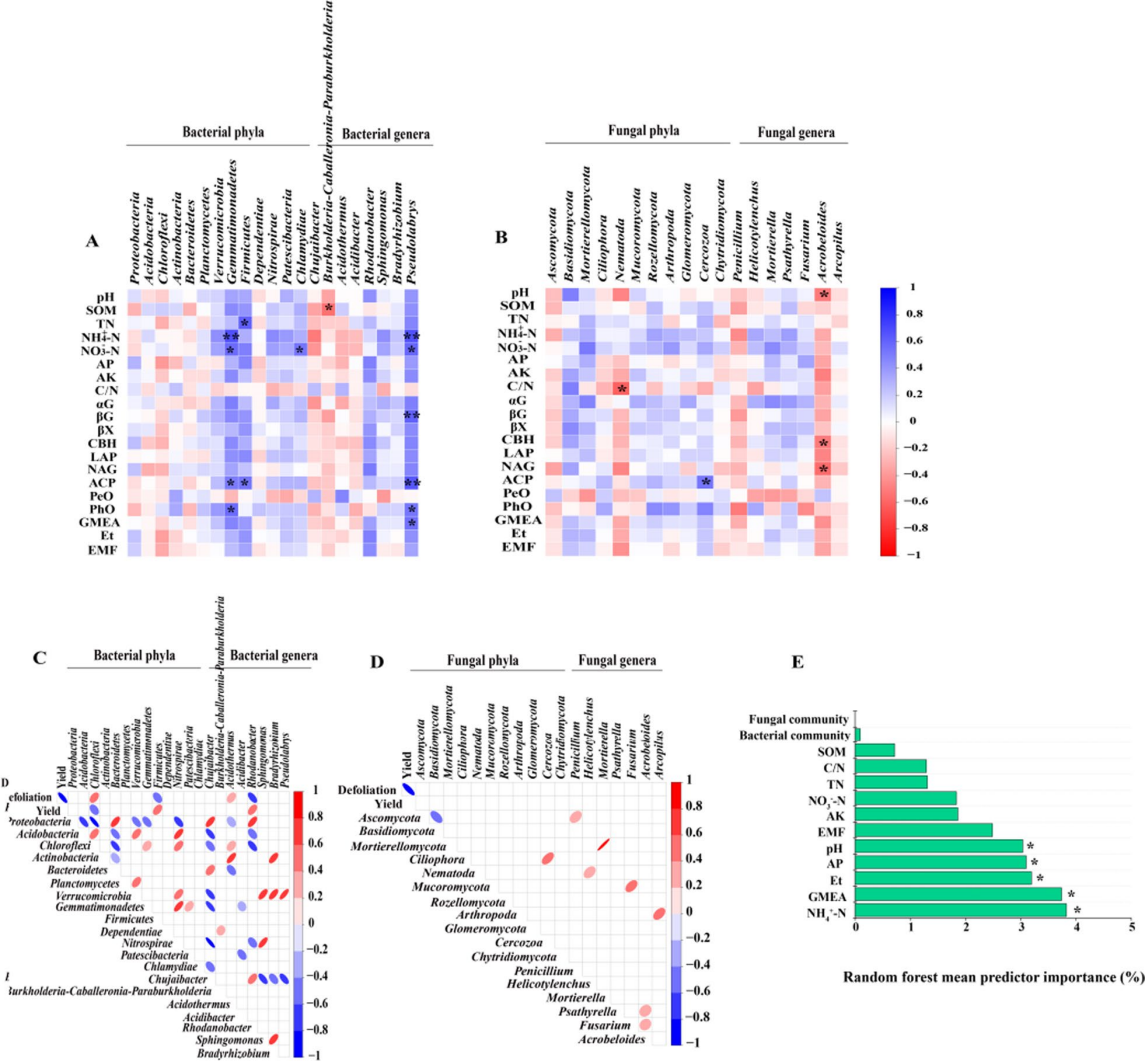
Spearman's correlation coefficients between soil physicochemical properties, enzyme activity, defoliation rate and the 15-dominant bacterial (**A**, **C**) and fungal taxa (**B**, **D**) of 2018–2019. Random forest mean predictor importance (% increase in MSE) of the soil physicochemical properties, microbial enzyme activities, and bacterial and fungal communities (NMDS1) with respect to early defoliation rate across all treatments (**E**). One and two asterisks represent significance at P < 0.05 and P < 0.01, respectively. Shades of blue and red represent a negative and positive correlation coefficient (r), respectively. Empty grids mean no difference. Significance levels of each predictor are as follows: *p < 0.05 and **p < 0.01

The relative abundance of *Gemmatimonadetes* showed significantly positive correlations with soil NH_4_^+^-N and NO_3_^−^-N, and *Firmicutes* significantly positively correlated with TN content (*P* < *0.05*). At the bacterial genus level, the relative abundance of *Burkholderia-Caballeronia-Paraburkholderia* significantly negative correlation with soil SOM. The relative abundance of *Pseudolabrys* significantly positive correlations with soil NH_4_^+^-N and NO_3_^−^-N. At the fungal genus level, the relative abundance of *Acrobeloides* significantly negative correlation with soil pH (Fig. 6A–B, P < *0.05*).

There are some relationships between soil enzyme activities and microbial taxa, such as βG, ACP, PhO and GMEA were all positively correlated with the relative abundance of *Pseudolabrys* at the bacterial genus level (*P* < *0.05*). CBH and NAG were significantly negatively correlated with the relative abundance of *Acrobeloides* at the fungal genus level (Fig. 6A–B, P < *0.05*).

The relative abundance of *Chloroflexi* showed a significantly negative correlation with early defoliation rate, but *Firmicutes* positively correlated with early defoliation rate (*P* < *0.05*). At the bacterial genus level, the relative abundances of *Acidothermus* and *Rhodanobacter* showed significantly negative and positive correlations with early defoliation rate (*P* < *0.05*), respectively. However, there are no significant correlations between fungal taxa and early defoliation rate (Fig. 6C–D). Additionally, random forest models identified that soil NH_4_^+^-N content as the main predictor of the early defoliation rate, followed by GMEA and Et. Additionally, the soil available phosphorus content and pH were also identified as the main predictors of the early defoliation rate (Fig. 6E).

## Discussion

### Effect of organic amendments on early defoliation rate

Soils in pear orchards in southern China, such as Fujian, Jiangxi and Zhejiang, are red loam, and the most suitable pH for pear tree growth should be between 5.5 and 6.5 (Wu et al. 2021; Zhao et al. 2011). Our results found that pH less than 5.0 in orchard soil treated by conventional fertilization (CK) (Table 1), indicating that the soil was troubled by acidification. Soil acidification usually inhibits the availability of phosphorus (P), calcium (Ca), iron (Fe) and other mineral nutrients (Guo et al. 2010; Hamilton et al. 2007). These changes may affect the accumulation of nutrient content in the leaves (Additional file 1: Table S3), thereby weakening the resistance of the pear leaves and causing a heavy early defoliation rate about 80% in CK treatment (Fig. 1A). Conversely, bioorganic fertilizer (BIO) and humic acid (HA) play an important role in alleviating soil acidification and increasing soil organic carbon, and humic acid (HA) also helps to stimulate the root growth and improve soil mineral nutrients availability. Both of them have been widely used to improve soil quality and increase fruit yield (Li et al. 2019b; Wang et al. 2017; Zaller 2006). In this study we found that addition of BIO and BIO-HA to the acid red soil of a sandy pear orchard significantly improved the soil pH to 5.3–5.5 (Table 1), which was close to the optimum condition for growth of pear trees in acid soil, indicating that organic amendments alleviated the soil acidification. Meanwhile, our results showed that organic amendments significantly inhibited the early defoliation rate of pear trees in acid soil (Fig. 1), which confirmed our hypothesis. These findings are similar to those reported by Rosmana et al. 2019, who found that application of organic fertilizers significantly reduced the early defoliation of cacao leaves. Spearman analysis showed that soil pH had a negative correlation with the early defoliation rate of pear trees, suggesting that application of organic amendments to alleviate soil acidification may be an important factor in the decrease of early defoliation rate of pear trees, which was supported by the result that soil pH was identified as one of the main predictors of the early defoliation rate based on the random forest models (Fig. 6).

Previous studies have found that a decrease in leaf N content and an increase in Ca, Fe, Mn, Cu and Zn content will reduce the occurrence of plant leaf diseases (Dordas 2008; Hoffland et al. 2000; Spann and Schumann 2009). Our results showed that BIO and BIO-HA treatments resulted in reduced leaf N content, and increased leaf Ca, Mg, Fe, Mn and Cu content (Additional file 1: Table S3). These results were supported by the significantly positive correlation between leaf N content and the early defoliation rate, and the significantly negative correlations between leaf Ca, Mg, Fe, Mn and Cu content and the early defoliation rate (Additional file 1: Fig. S1), indicating that the application of bioorganic fertilizer or the combination of bioorganic fertilizer and humic acid changes the nutrient status of sandy pear leaves to enhance resistance to biotic or abiotic stress (e.g. pathogen infection), thereby reducing the early defoliation rate. Besides, the increased in leaf mineral nutrients with the application of BIO and BIO-HA may be related to the alleviation of the soil acidification, which will increase the availability of mineral nutrients in orchard soil. This was supported by the significant correlations between soil pH and leaf medium-trace nutrient content (Additional file 1: Fig. S1). Additionally, we found that the pear yield treated with BIO or BIO-HA was 1.3 times higher than that of the CK and CK-HA treated trees, and that yield was strongly negatively correlated with the early defoliation rate (Fig. 1 and Additional file 1: Fig. S1), indicating that organic amendments alleviated the early defoliation rate of sandy pear is an important factor in the increasing yield.

### Effect of organic amendments on soil enzymeactivity

It has been highlighted in many studies that soil pH was an important driver of microbial activity, diversity and ecosystem multifunctionality (EMF) (Luo et al. 2018; Xun et al. 2016). In the present study, we found that there were significant relationships between soil pH and total enzyme activity and EMF (Additional file 1: Fig. S1), indicating that application of BIO and BIO-HA alleviated the soil acidification was an important factor in improving soil enzyme activity and EMF. Previous studies have demonstrated that using organic amendments to improve soil enzyme activity and EMF is considered an important strategy for increasing crop yields (Li et al. 2019a; Tamburini et al. 2020; Wang et al. 2016). Our results show that BIO and BIO-HA treatments significantly increased all enzyme activities related to the carbon, nitrogen and phosphorus cycles after 3-year fertilization, except PeO and PhO (Additional file 1: Fig. S3), which was generally consistent with the results of previous studies (Luo et al. 2018; Wang et al. 2016). We also found that BIO and BIO-HA treatments significantly increased Et and EMF (Fig. 1), which are in agreement with previous studies (Luo et al. 2018; Wang et al. 2016), and we found pear yield from BIO and BIO-HA treated trees was positively correlated with Et and EMF (Additional file 1: Fig. S1). Thus, organic amendments would be essential to improve soil fertility to obtain the increasing in fruit yield. Soil fertility directly affects the growth and health of plants. Interestingly, we found that the decrease in the early defoliation rate of sandy pear trees treated with organic amendments was related to the significant increase in soil Et and EMF (Additional file 1: Fig. S1). This was partially consistent with the result that Et can be used as an important predictor of early defoliation rate by using the random forest model (Fig. 6). Our results demonstrate that application of organic amendments into the acid red soil of the sandy pear orchard increased soil enzyme activities, which is an important factor in alleviating the early defoliation rate of sandy pear trees.

### Effect of organic amendments on soil microbial communities and diversity

We found that the application of organic amendments, for three consecutive years, did not significantly impact the soil microbial diversity of the orchard soil (Additional file 1: Fig. S4), but did significantly change the soil bacterial and fungal communities (Fig. 2). This is like results from previous studies (Li et al. 2019b; Liu et al. 2020b; Wang et al. 2016). At the phylum level, we found that *Proteobacteria* and *Acidobacteria* were the dominant bacteria, and *Ascomycota* and *Basidiomycota* were the dominant fungi in the acid red soil of pear orchard across all treatments (Fig. 3). This is also consistent with the findings of other studies (Gu et al. 2019; Li et al. 2019b; Liu et al. 2020b). Here, we found that the relative abundance of *Proteobacteria*, *Bacteroidetes*, *Firmicutes*, and *Nitrospirae* in the bacterial taxa, and *Basidiomycota* and *Mortierellomycota* in the fungal taxa increased after application of BIO and HA for three years (Fig. 3). These microorganisms have been shown to promote plant growth (Lee et al. 2021; Mendes et al. 2011; Wang et al. 2016; Wu et al. 2011). The results of our present study has confirmed that the use of BIO and HA reduces the relative abundance of the *Acidobacteria* and *Chloroflexi* in bacterial taxa, which is consistent with the results of previous studies (Li et al. 2019b; Liu et al. 2019; Wang et al. 2016). The response of *Ascomycota* (most of which are pathogenic to plants) (Xu et al. 2012) to organic amendments varies depending on crop type. For example, Wang et al. (2016) and Liu et al. (2020b) found that bioorganic fertilizer increased the relative abundance of *Ascomycota* in apple and kiwi orchard soils, while Li et al. (2019a, b) found that the application of humic acid reduced the relative abundance of *Ascomycota* in peanut-growing soils. We have demonstrated in our study that applications of BIO and HA resulted in the reduced relative abundance of Ascomycota. Our data demonstrates that bioorganic fertilizer and humic acid inhibits fungal diseases and promotes tree health when applied to the acid red soil of sandy pear orchards.

*Firmicutes* have an important role in stimulating plants’ systemic resistance to disease (Lee et al. 2021; Mendes et al. 2011). Our study demonstrated that the relative abundance of *Firmicutes* in soil was significantly negatively correlated with the early defoliation rate of sandy pear trees (Fig. 6). The result suggests that the changes in soil properties from BIO and HA application allow for the accumulation of *Firmicutes*, which may in turn stimulate the disease resistance of sandy pear trees, and their leaves, thus decreasing the early defoliation rate. Additionally, Kang et al. (2016) found that accumulation of *Firmicutes* on the surface of plant leaves is closely related to a reduction of leaf diseases, highlighting that the interaction between phyllospheric microorganisms and pathogens in sandy pear leaves deserves future research.

*Acidothermus* are generally enriched in soils with severe disease (Gao et al. 2020; Svenningsen et al. 2018). *Rhodanobacter* have an antagonistic effect on fungal pathogens in soil (De Clercq et al. 2006; Huang et al. 2016). We observed that organic amendments reduced the relative abundance of *Acidothermus* in sandy pear orchard soil and increased the relative abundance of *Rhodanobacter*. The increase in the relative abundance of *Rhodanobacter* may inhibit the fungal pathogens (e.g., *Colletotrichum*) parasitic on leaf litter in the soil, in turn reducing the early defoliation rate of sandy pear trees. This is supported by the significantly negative correlation between the relative abundance of *Rhodanobacter* and the early defoliation rate (Fig. 6). However, the effect of organic amendments on the dominant fungal taxa in acid red soil of sandy pear orchards has no obvious correlation with the early defoliation rate (Fig. 6). This was consistent with the result that the fungal community wasn’t as a significant predictor for the early defoliation rate based on the random forest model analysis (Fig. 6), indicating that the fungal community may indirectly affect the aboveground growth of pear trees through the interaction with the bacterial community.

### Effect of organic amendments on soil microbial co-occurrence networks

When the composition of soil microbial communities change, as they do when fertilization practices change, the microbial co-occurrence network changes as well (Ling et al. 2016; Ramirez et al. 2018). Network topology parameters have been used as biological indicators of the adaptability of microorganisms in response to fertilization disturbances (Ji et al. 2020; Li et al. 2020a; Liu et al. 2020a). In our field test, we found that application of BIO (+ BIO) and HA (+ HA) reduced the modularity of the co-occurrence network of bacteria and fungi (Fig. 5, Additional file 1: Fig. S6 and Table S4), this is consistent with previous research results of Liu et al. 2020c. Complex networks with higher connectivity are more robust to environmental disturbances than simple networks with lower connectivity (Santolini and Barabási 2018), and a large number of studies have shown that organic amendments increase the complexity of the microbial co-occurrence network in soils (Gu et al. 2019; Liu et al. 2020c; Price et al. 2021). The results of our study showed that the application of + BIO (vs -BIO) for 3 consecutive years had little effect on the number of edges and network density of the co-occurrence network of soil bacteria and fungi, but the clustering coefficient and average path length was reduced. These results mean that the application of BIO improved the efficiency of the co-occurrence network to quickly respond to environmental changes, it does not increase the complexity of the co-occurrence network in the sandy pear orchard soil (Liu et al. 2020c; Zhou et al. 2010). Unlike + BIO, + HA addition (vs -HA) did increase the complexity of the whole network and improve its efficiency (Liu et al. 2020c; Zhou et al. 2010). In sum, organic amendments increase the abundance of beneficial microbial functional taxa in the acid red soil of southern China’s sandy pear orchards, which may be correlated to the decrease in the early defoliation rate of pear trees.

## Supplementary Information

**Additional file 1: Table S1.** Fertilization protocol from 2017 to 2019. CK, conventional fertilization; CK-HA, conventional fertilization combined with humic acid (HA); BIO, bio-organic fertilizer; BIO-HA, mixed application of BIO and HA. **Table S2.** Extracellular enzymes with corresponding commission number (EC), corresponding substrate, and the abbreviation used in this study. **Table S3.** Effect of organic amendments on the leaf nutrient concentrations of sandy pear. Each value represents the mean (n = 6), and standard error values are indicated with ± . Different letters indicate significant difference (*P* < *0.05*) in every row among the four treatments as determined by Fisher’s least significant difference test (LSD) at α = 0.05. **Table S4.** Network topological characteristics calculated by Network Analyzer tool in Gephi 0.9.2. **Figure S1.** Spearman correlations show the relationships among different functional groups. Shades of blue and red represent a negative and positive correlation coefficient (r), respectively. Empty grids mean no difference. The detailed traits of the different functional groups can be found in the data analysis section described in the article. **Figure S2.** Effects of organic amendments on (A) SPAD, (B) leaf area and (C) leaf thickness. Each value represents the mean (n = 6), and the error bars are the standard errors. Significant differences are indicated by different lowercase letters at *P* < *0.05* based on the LSD test. **Figure S3.** Nutrient-cycle enzyme activities across experimental treatments. αG, α-1,4-Glucosidase; βG, β-1,4-Glucosidase; βX, β-1,4-Xylosidase; CBH, β-D-Cellobiohydrolase; LAP, Leucine amino peptidase; NAG, β-1,4-N-Acetyl-glucosaminidase; ACP, Acid phosphomonoesterase; PeO, Peroxidas; PhO, Phenol oxidase. Different lowercase letters indicate significant difference at 0.05 levels (LSD, *P* < *0.05*) among different fertilizer treatments. The error bars are the standard errors (n = 6). **Figure S4.** Bacterial (A and B) and fungal (C and D) diversity by fertilization treatment 2018 to 2019. Different lowercase letters indicate significant difference at 0.05 levels (LSD, *P* < *0.05*) among different treatments. The error bars are the standard errors (n = 6). **Figure S5.** Significance analysis of the relative abundances of the 15-dominant bacterial (A) and fungal (B) phyla based on the LSD at 0.05 levels in pear orchard soils in 2018—2019. * indicating that there are significant differences between CK and CK-HA, BIO and BIO-HA, respectively. **Figure S6.** Network analysis revealing the co-occurrence pattern between bacterial and fungal OTUs in -HA (CK and BIO) and + HA (CK-HA and BIO-HA) treated soils, 2018 and 2019. Colored nodes signify corresponding OTUs assigned to major phyla. Red line and blue line represent strong positive linear (r > 0.6) and strong negative linear (r < -0.6) and relationships, respectively. The size of each node is proportional to the number of connections (degree).

## Data Availability

All data generated or analyzed during this study are included in this published article (and its additional file).

## Abbreviations

BIO: Bioorganic fertilizer
HA: Humic acid
NMDS: Non-metric multidimensional scaling
ED: Early defoliation rate
SPAD: Relative chlorophyll content
LA: Leaf area
SOM: Soil organic matter
αG: α-1,4-Glucosidase
βG: β-1,4-Glucosidase
CBH: β-D-Cellobiohydrolase
LAP: Leucine amino peptidase
NAG: β-1,4-N-Acetyl-glucosaminidase
ACP: Acid phosphomonoesterase
PhO: Phenol oxidase
PeO: Peroxidas
GMEA: Geometric mean enzyme activity
Et: Total enzyme activity
EMF: Ecosystem multifunctionality
PERMANOVA: Permutation multivariate analysis of variance
ANOSIM: Similarity analysis
