# Supplementary material for: Organic amendments alleviate early defoliation and increase fruit yield by altering assembly patterns and of microbial communities and enzymatic activities in sandy pear (Pyrus pyrifolia)
Source: AMB Express. 2021 Dec 8;11:164. doi: 10.1186/s13568-021-01322-5 (PMC8655061; doi:10.1186/s13568-021-01322-5)

**Supplementary materials**

**Title: Organic amendments alleviate early defoliation and increase fruit yield by altering assembly patterns and of microbial communities and enzymatic activities in sandy pear (*Pyrus pyrifolia*)**

**Journal name:** AMB express

**Authors and addresses:** Yalong KANG^a^, Xiangrui AN^a^, Yanwei MA^a^, Shaomin ZENG^b^, Shangtao JIANG^a^, Wenli WU^a^, Changyan XIE^c^, Zhonghua WANG^d^, Caixia DONG^a*^, Yangchun XU^a^, Qirong SHEN^a^

a. Jiangsu Provincial Key Lab for Organic Solid Waste Utilization, National Engineering Research Center for Organic-based Fertilizers, Jiangsu Collaborative Innovation Center for Solid Organic Waster Resource Utilization, Nanjing Agricultural University, Nanjing 210095, China

b. Fruit Research Institute，Fujian Academy of Agricultural Sciences, Research Centre for Engineering Technology of Fujian Deciduous Fruits, Fuzhou 350013, China

c. The research center of agricultural resources environment and soil fertilizer, Huaian academy of agricultural sciences, Huaian 223001, China

d. Institute of Pomology, Jiangsu Academy of Agricultural Sciences, Nanjing 210014, China

* Corresponding author: Dr. Caixia Dong. Email: [cxdong@njau.edu.cn](mailto:cxdong@njau.edu.cn)

**Supplementary Table S1** Fertilization protocol, 2017 to 2019.

| Treatments | Base fertilizer | | | | Topdressing |
| --- | --- | --- | --- | --- | --- |
|  | BIO  kg hm^-1^ | HA  kg hm^-1^ | Conventional organic fertilizer  kg hm^-1^ | Compound fertilizer  kg hm^-1^ | Compound fertilizer  kg hm^-1^ |
| CK | 0 | 0 | 6,000 | 4,800 | 1,200 |
| CK-HA | 0 | 240 | 6,000 | 4,800 | 1,200 |
| BIO | 12,000 | 0 | 0 | 0 | 0 |
| BIO-HA | 12,000 | 240 | 0 | 0 | 0 |

CK, conventional fertilization; CK-HA, conventional fertilization combined with humic acid (HA); BIO, bio-organic fertilizer; BIO-HA, mixed application of BIO and HA. compound fertilizer (N-P_2_O_5_-K_2_O, 24:8:10); bioorganic fertilizer (N+P_2_O_5_+K_2_O ≥ 8%, organic content ≥ 40%, pH 6.6); conventional organic fertilizer (N+P_2_O_5_+K_2_O ≥ 5%, organic content ≥ 50%); humic acid (humic acid 30%, N+P_2_O_5_+K_2_O = 12:5:3, pH 6.0).

**Supplementary Table S2.** Extracellular enzymes with corresponding commission number (EC), corresponding substrate, and the abbreviation used in this study.

| Cor. Cycle^1^ | Enzyme | Abbreviation | Substrate | EC |
| --- | --- | --- | --- | --- |
| C-cycling  N-cycling | Peroxidas | PeO | L-DOPA^3^ | 1.11.1.7 |
|  | Phenol oxidase | PhO | L-DOPA | 1.10.3.2 |
|  | α-1,4-Glucosidase | αG | 4-MUB^2^-α-D-glucoside | 3.2.1.20 |
|  | β-1,4-Glucosidase | βG | 4-MUB-β-D-glucoside | 3.2.1.21 |
|  | β-1,4-xylosidase | βX | 4-MUB-β-D-xyloside | 3.1.1.37 |
|  | β-D-Cellobiohydrolase | CBH | 4-MUB-β-D-cellobioside | 3.2.1.91 |
|  | Leucine amino peptidase | LAP | L-Leucine-7-amino-4-methylcoumarin | 3.4.11.1 |
|  | β-1,4-N-Acetyl-glucosaminidase | NAG | 4-MUB-N-acetyl-β-D-glucosaminide | 3.2.1.30 |
| P-cycling | Acid phosphomonoesterase | ACP | 4-MUB-phosphate | 3.1.3.2 |

^1^ The enzymes involved in this cycling process

^2^ 4-MUB, 4-methylumbelliferyl
^3^ L-DOPA, L-3,4-dihydroxyphenylalanine

**Supplementary Table S3.** Effect of organic amendments on sandy pear leaf nutrient concentrations.

| Leaf nutrient concentrations in 2018 | CK | CK-HA | BIO | BIO-HA |
| --- | --- | --- | --- | --- |
| C g kg^-1^ | 236.3±25.04 | 259.1±6.83 | 295.1±34.99 | 272.3±10.33 |
| N g kg^-1^ | 22.6±2.03a | 20.2±2.11ab | 15.6±0.91b | 19.3±0.31ab |
| P g kg^-1^ | 1.5±0.08 | 1.8±0.17 | 1.6±0.2 | 1.9±0.24ns |
| K g kg^-1^ | 20.1±0.55b | 22.3±2.50ab | 30.4±2.17a | 29.7±1.96a |
| Ca g kg^-1^ | 18.1±1.24b | 23.2±2.98b | 33.3±0.63a | 32.1±0.81a |
| Mg g kg^-1^ | 2.3±0.05 | 2.4±0.27 | 2.8±0.35 | 3.2±0.14ns |
| Fe mg kg^-1^ | 92.6±2.96b | 113.9±10.09b | 148.9±27.37ab | 192.9±1.71a |
| Mn mg kg^-1^ | 92.7±9.49b | 118.2±11.79ab | 142.2±14.27ab | 167.1±15.19a |
| Cu mg kg^-1^ | 9.4±1.22b | 12.4±1.73b | 18.2±0.32a | 22.4±1.16a |
| Zn mg kg^-1^ | 89.1±6.42 | 96.3±1.65 | 110.3±9.46 | 108.3±3.49ns |
| Leaf nutrient concentrations in 2019 | CK | CK-HA | BIO | BIO-HA |
| C g kg^-1^ | 236.6±1.61 | 235.6±2.47 | 257.6±8.02 | 252.3±10.69 |
| N g kg^-1^ | 20.5±0.55a | 18.7±1.13ab | 15.1±0.94b | 15.9±1.16b |
| P g kg^-1^ | 1.5±0.07 | 1.7±0.14 | 1.5±0.06 | 1.8±0.01ns |
| K g kg^-1^ | 19.5±1.05 | 21.7±2.47 | 26.3±2.22 | 28±2.14ns |
| Ca g kg^-1^ | 19±2.25c | 24.7±0.56b | 28.8±0.52ab | 30.2±0.98a |
| Mg g kg^-1^ | 2.5±0.16b | 2.5±0.13b | 2.8±0.07ab | 3.3±0.16a |
| Fe mg kg^-1^ | 85.5±3.61b | 101.8±6.96b | 158.9±20.47a | 201.5±17.79a |
| Mn mg kg^-1^ | 96.5±13.53b | 105.5±7.84b | 154.5±17.23ab | 184.7±14.58a |
| Cu mg kg^-1^ | 9.6±0.76b | 13.2±1.96b | 21.4±1.82a | 20.8±0.58a |
| Zn mg kg^-1^ | 85.4±4.55 | 93.2±4.86 | 104.1±4.84 | 112.7±10.89ns |

CK, conventional fertilization; HA, humic acid amendment; CK-HA, conventional fertilization combined with HA; BIO, bio-organic fertilizer; BIO-HA, mixed application of BIO and HA. Each value represents the mean (n = 6), and standard error values are indicated with ±. Different letters indicate significant difference (*P < 0.05*) in every row among the four treatments as determined by Fisher’s least significant difference test (LSD) at α = 0.05. The ns after the maximum indicates that there is no significant difference between treatments.

**Supplementary Table S4.** Network topological characteristics calculated by Network Analyzer tool in Gephi 0.9.2.

| Bacterial and Fungal taxa | Network topological characteristics in 2018 | -BIO | | +BIO | | -HA | | +HA |
| --- | --- | --- | --- | --- | --- | --- | --- | --- |
|  | Number of nodes | 127 | 127 | | 127 | | 127 | |
|  | Bacterial nodes (%) | 51 | 51 | | 51 | | 51 | |
|  | Fungal nodes (%) | 49 | 49 | | 49 | | 49 | |
|  | Number of positive edges in bacterial taxa | 259 | 279 | | 128 | | 228 | |
|  | Number of negative edges in bacterial taxa | 88 | 86 | | 38 | | 51 | |
|  | Number of positive edges between bacteria and fungi | 434 | 401 | | 197 | | 305 | |
|  | Number of negative edges between bacteria and fungi | 53 | 77 | | 79 | | 32 | |
|  | Number of positive edges in fungal taxa | 278 | 305 | | 218 | | 200 | |
|  | Number of negative edges in fungal taxa | 8 | 9 | | 7 | | 1 | |
|  | Number of all edges | 1120 | 1157 | | 667 | | 817 | |
|  | Network density | 0.140 | 0.144 | | 0.083 | | 0.102 | |
|  | Clustering coefficient | 0.709 | 0.694 | | 0.537 | | 0.681 | |
|  | Average path length | 3.000 | 2.763 | | 3.318 | | 3.142 | |
|  | Network diameter | 8 | 6 | | 8 | | 7 | |
|  | Network centralization | 0.178 | 0.222 | | 0.097 | | 0.097 | |
|  | Modularity (no. of modules) | 0.494 (6) | 0.483 (8) | | 0.616 (7) | | 0.649 (8) | |
| Bacterial and Fungal taxa | Network topological characteristics in 2019 | -BIO | | +BIO | | -HA | | +HA |
|  | Number of nodes | 187 | 187 | | 187 | | 187 | |
|  | Bacterial nodes (%) | 51 | 51 | | 51 | | 51 | |
|  | Fungal nodes (%) | 49 | 49 | | 49 | | 49 | |
|  | Number of positive edges in bacterial taxa | 421 | 435 | | 332 | | 385 | |
|  | Number of negative edges in bacterial taxa | 19 | 49 | | 60 | | 28 | |
|  | Number of positive edges between bacteria and fungi | 656 | 644 | | 610 | | 683 | |
|  | Number of negative edges between bacteria and fungi | 14 | 43 | | 62 | | 24 | |
|  | Number of positive edges in fungal taxa | 487 | 414 | | 419 | | 477 | |
|  | Number of negative edges in fungal taxa | 12 | 14 | | 28 | | 6 | |
|  | Number of all edges | 1609 | 1600 | | 1511 | | 1603 | |
|  | Network density | 0.093 | 0.093 | | 0.083 | | 0.092 | |
|  | Clustering coefficient | 0.729 | 0.713 | | 0.686 | | 0.726 | |
|  | Average path length | 3.843 | 3.353 | | 3.545 | | 3.538 | |
|  | Network diameter | 9 | 7 | | 8 | | 7 | |
|  | Network centralization | 0.116 | 0.142 | | 0.175 | | 0.109 | |
|  | Modularity (no. of modules) | 0.750 (8) | 0.738 (8) | | 0.757 (8) | | 0.744 (7) | |

**Fig. S1** Spearman correlations show the relationships among different functional groups. Shades of blue and red represent a negative and positive correlation coefficient (r), respectively. Empty grids mean no difference. The detailed traits of the different functional groups can be found in the data analysis section described in the article.


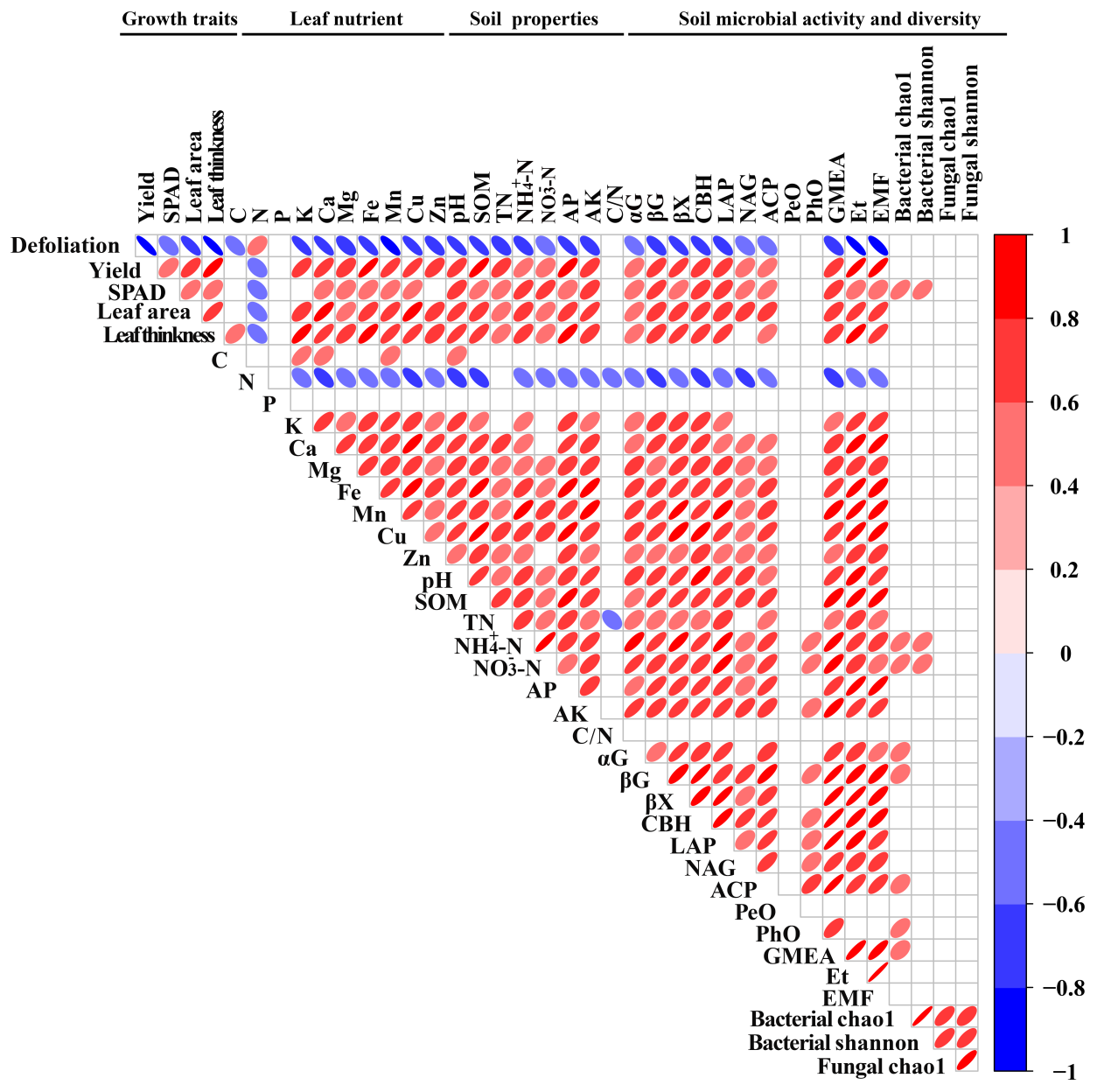


**Fig. S2** Effects of organic amendments on (A) SPAD, (B) leaf area and (C) leaf thickness. CK, conventional fertilization; HA, humic acid amendment; CK-HA, conventional fertilization combined with HA; BIO, bio-organic fertilizer; BIO-HA, mixed application of BIO and HA. Each value represents the mean (n = 6), and the error bars are the standard errors. Significant differences are indicated by different lowercase letters at *P < 0.05* based on the LSD test.


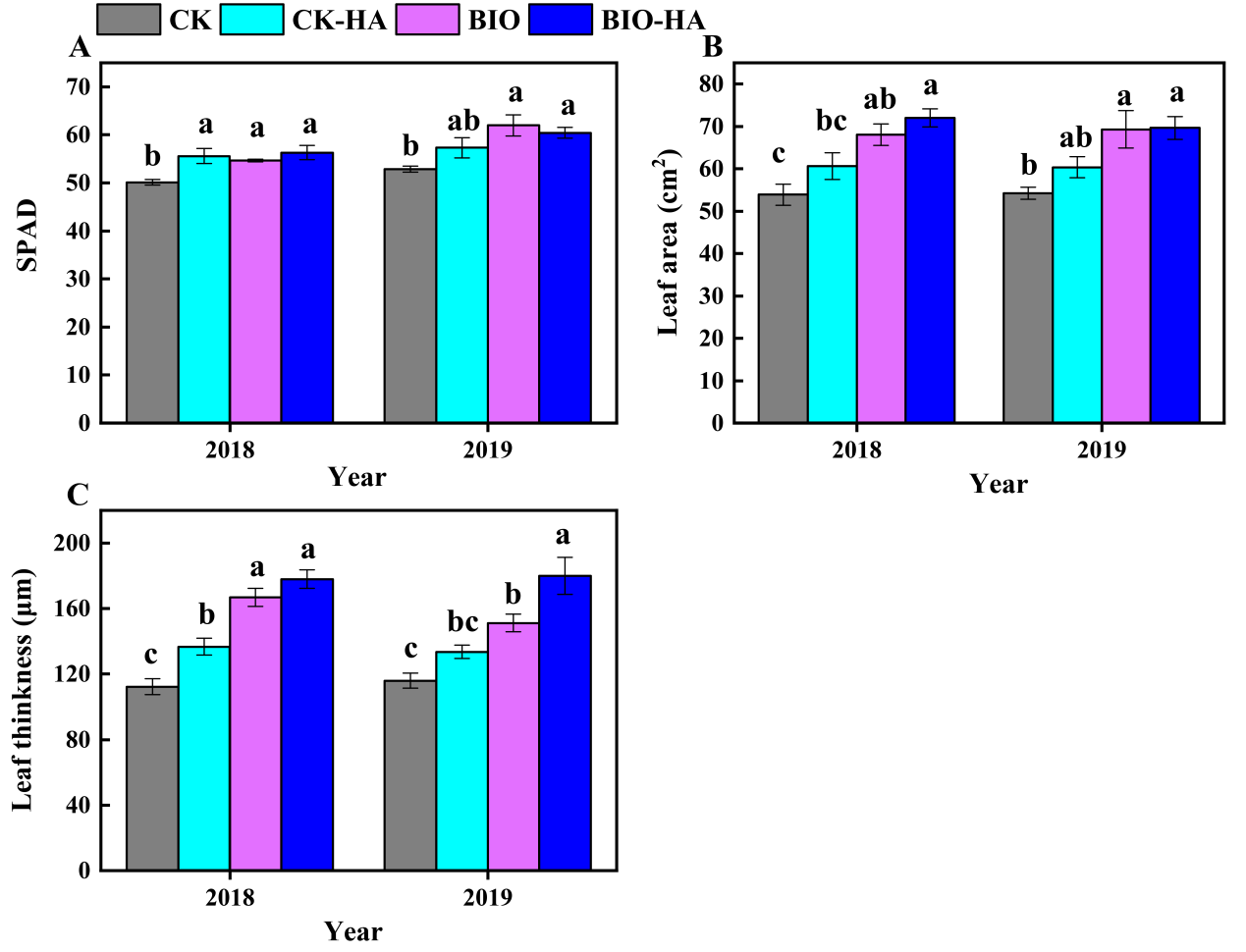


**Fig. S3** Nutrient-cycle enzyme activities across experimental treatments. αG, α-1,4-Glucosidase; βG, β-1,4-Glucosidase; βX, β-1,4-Xylosidase; CBH, β-D-Cellobiohydrolase; LAP, Leucine amino peptidase; NAG, β-1,4-N-Acetyl-glucosaminidase; ACP, Acid phosphomonoesterase; PeO, Peroxidas; PhO, Phenol oxidase; CK, conventional fertilization; HA, humic acid amendment; CK-HA, conventional fertilization combined with HA; BIO, bio-organic fertilizer; BIO-HA, mixed application of BIO and HA. Different lowercase letters indicate significant difference at 0.05 levels (LSD, *P < 0.05*) among different fertilizer treatments. The error bars are the standard errors (n = 6).


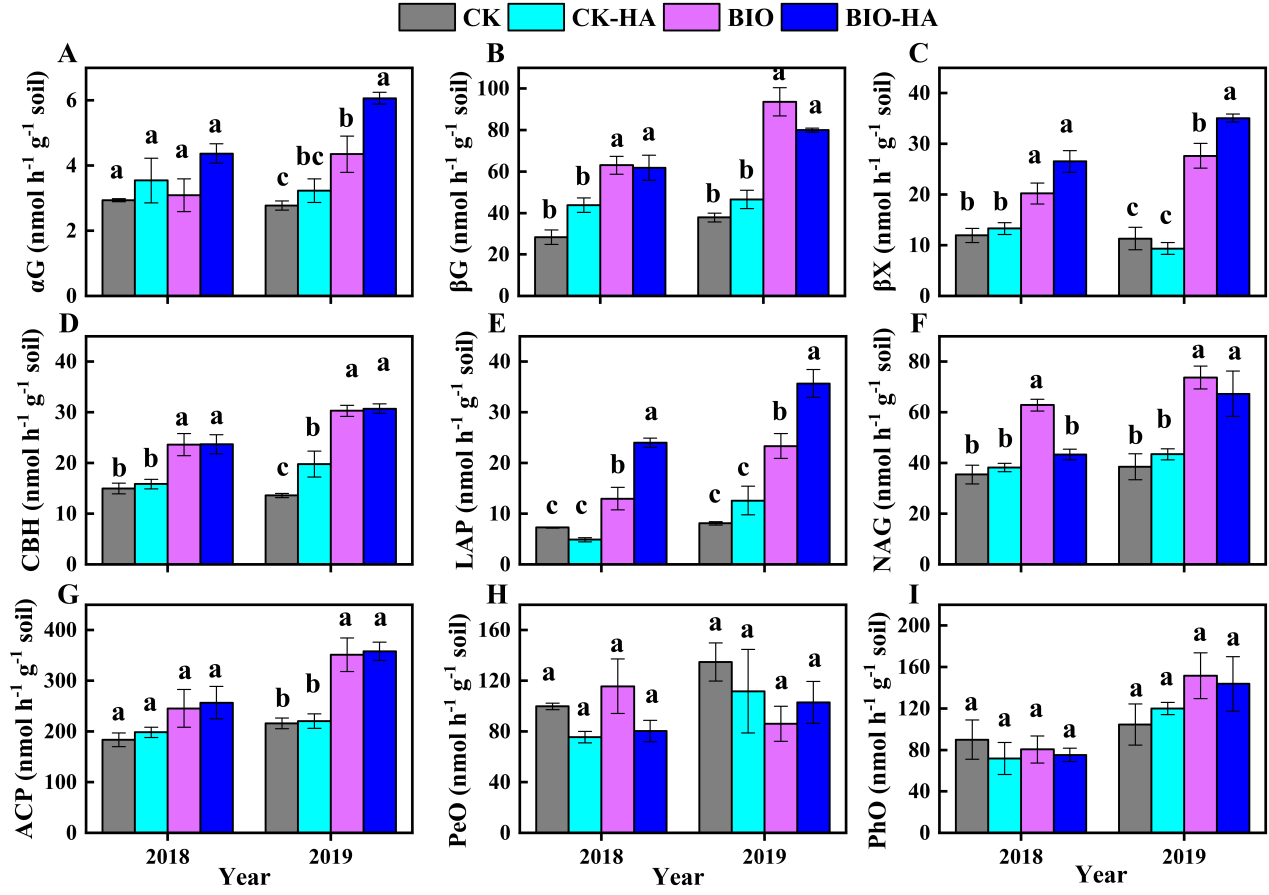


**Fig. S4** Bacterial (A and B) and fungal (C and D) diversity by fertilization treatment 2018 to 2019. CK, conventional fertilization; HA, humic acid amendment; CK-HA, conventional fertilization combined with humic acid amendment; BIO, bio-organic fertilizer; BIO-HA, combined application of BIO and HA. Different lowercase letters indicate significant difference at 0.05 levels (LSD, *P < 0.05*) among different treatments. The error bars are the standard errors (n = 6).


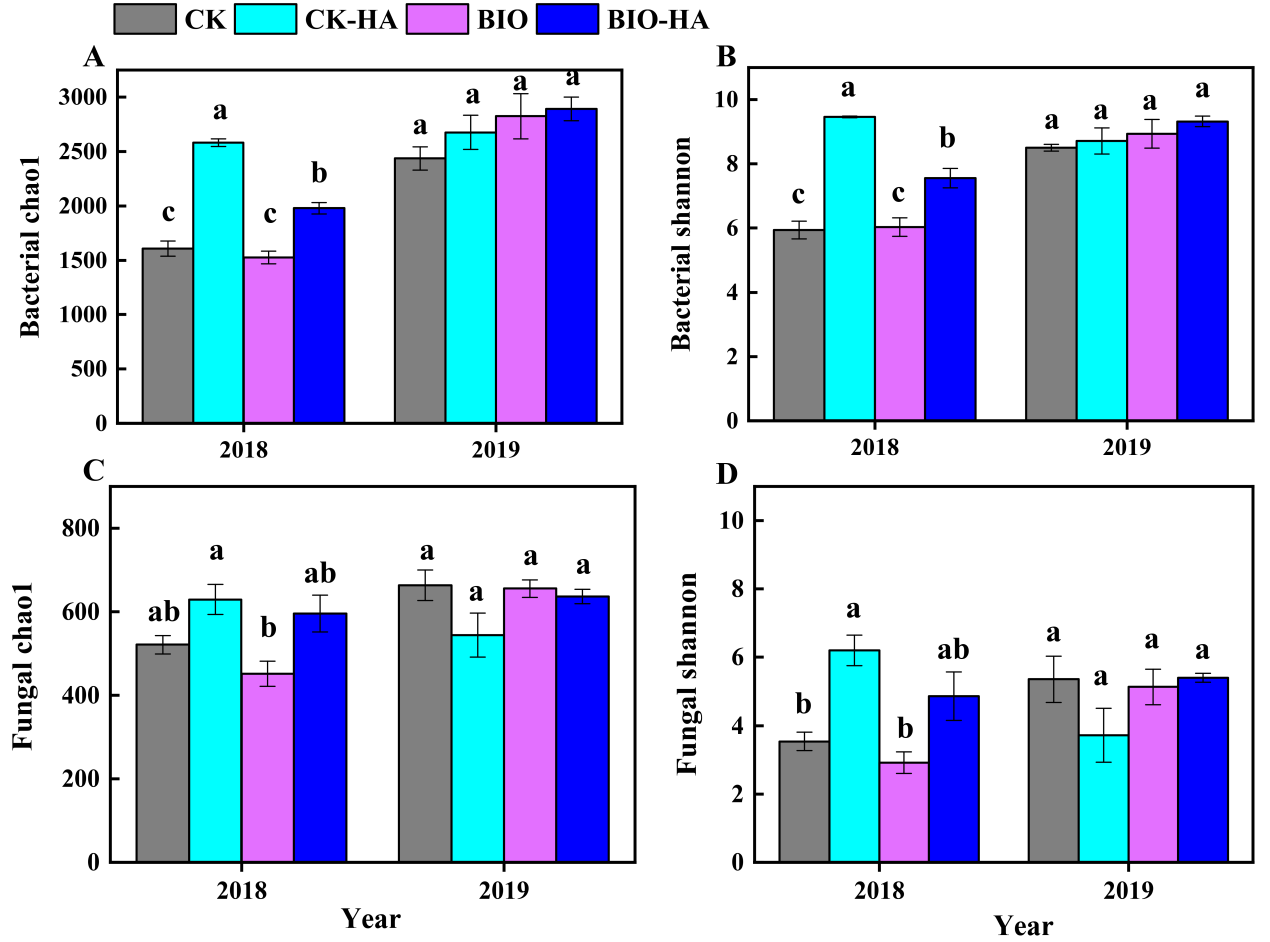


**Fig. S5** Significance analysis of the relative abundances of the 15-dominant bacterial (A) and fungal (B) phyla based on the LSD at 0.05 levels in pear orchard soils in 2018 - 2019. * indicating that there are significant differences between CK and CK-HA, BIO and BIO-HA, respectively.


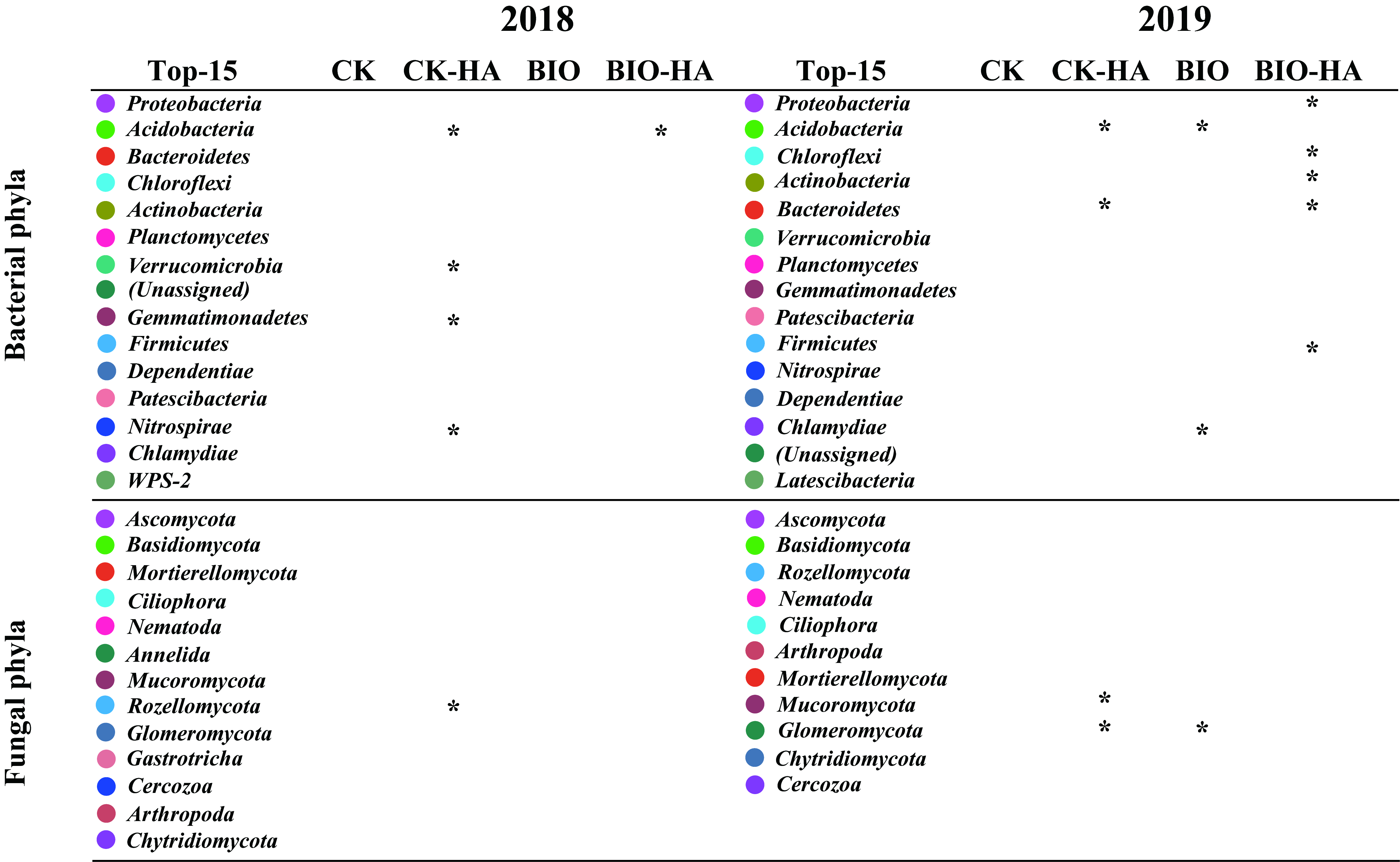


**Fig. S6** Network analysis revealing the co-occurrence pattern between bacterial and fungal OTUs in -HA (CK and BIO) and +HA (CK-HA and BIO-HA) treated soils, 2018 and 2019. Green and black colored nodes signify corresponding OTUs assigned to bacterial and fungal major phyla, respectively. Red line and blue line represent strong positive linear (r > 0.6) and strong negative linear (r < -0.6) and relationships, respectively. The size of each node is proportional to the number of connections (degree).


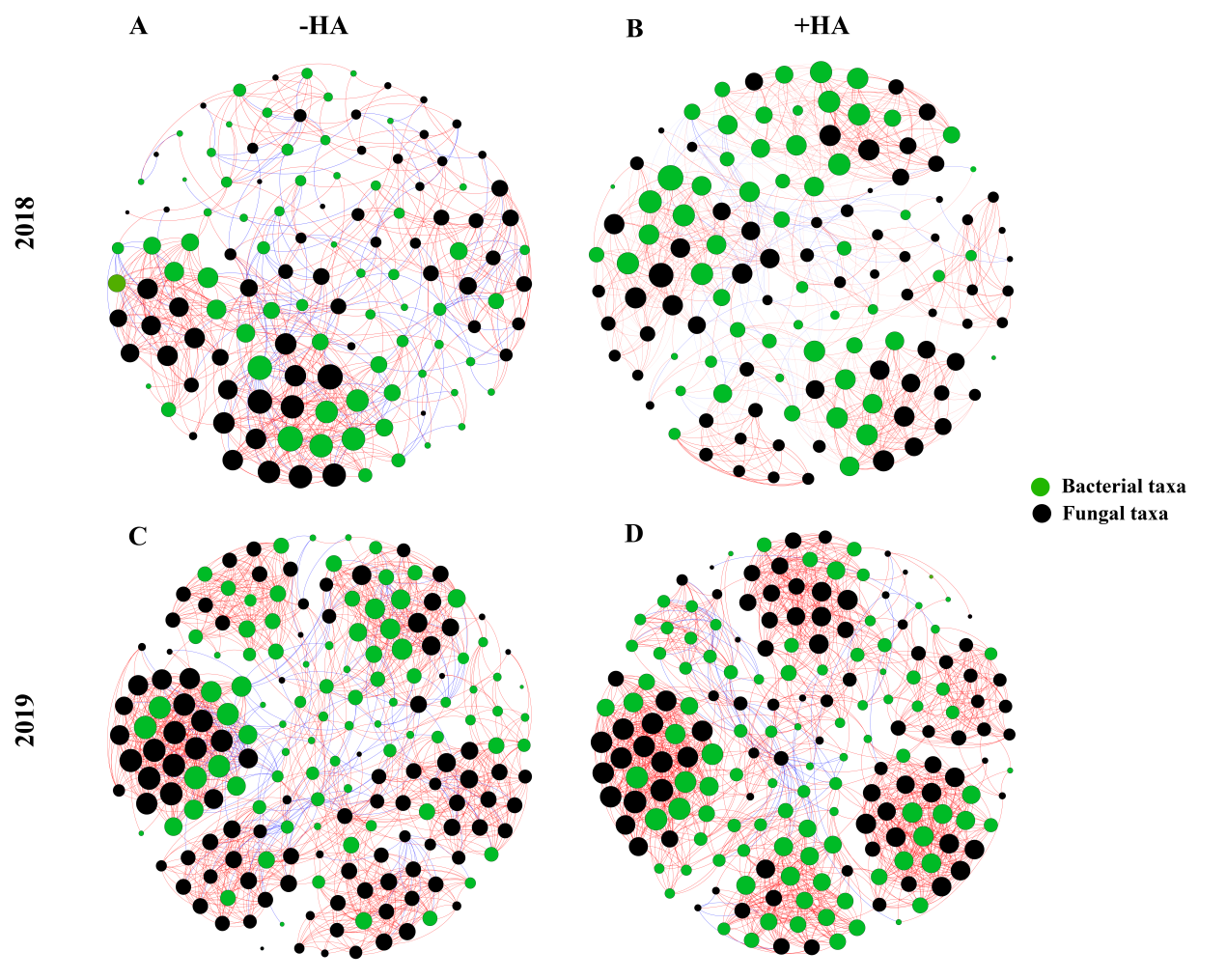

Supplement: Supplementary file 1 — Additional file 1: Table S1. Fertilization protocol from 2017 to 2019. CK, conventional fertilization; CK-HA, conventional fertilization combined with humic acid (HA); BIO, bio-organic fertilizer; BIO-HA, mixed application of BIO and HA. Table S2. Extracellular enzymes with corresponding commission number (EC), corresponding substrate, and the abbreviation used in this study. Table S3. Effect of organic amendments on the leaf nutrient concentrations of sandy pear. Each value represents the mean (n = 6), and standard error values are indicated with ± . Different letters indicate significant difference (P < 0.05) in every row among the four treatments as determined by Fisher’s least significant difference test (LSD) at α = 0.05. Table S4. Network topological characteristics calculated by Network Analyzer tool in Gephi 0.9.2. Figure S1. Spearman correlations show the relationships among different functional groups. Shades of blue and red represent a negative and positive correlation coefficient (r), respectively. Empty grids mean no difference. The detailed traits of the different functional groups can be found in the data analysis section described in the article. Figure S2. Effects of organic amendments on (A) SPAD, (B) leaf area and (C) leaf thickness. Each value represents the mean (n = 6), and the error bars are the standard errors. Significant differences are indicated by different lowercase letters at P < 0.05 based on the LSD test. Figure S3. Nutrient-cycle enzyme activities across experimental treatments. αG, α-1,4-Glucosidase; βG, β-1,4-Glucosidase; βX, β-1,4-Xylosidase; CBH, β-D-Cellobiohydrolase; LAP, Leucine amino peptidase; NAG, β-1,4-N-Acetyl-glucosaminidase; ACP, Acid phosphomonoesterase; PeO, Peroxidas; PhO, Phenol oxidase. Different lowercase letters indicate significant difference at 0.05 levels (LSD, P < 0.05) among different fertilizer treatments. The error bars are the standard errors (n = 6). Figure S4. Bacterial (A and B) and fungal (C and D) di [file 13568_2021_1322_MOESM1_ESM.docx]
